# Chromatographic‐Based Binding and Thermodynamic Studies of Antibiotic Micropollutants with Humic Acid Using Affinity Microcolumns

**DOI:** 10.1002/jssc.70345

**Published:** 2026-01-13

**Authors:** Sadia Sharmeen, Isaac Kyei, Saumen Poddar, Sazia Iftekhar, BK Sajeeb, Lillian M. Graham, Daniel D. Snow, David S. Hage

**Affiliations:** ^1^ Department of Chemistry University of Nebraska‐Lincoln Lincoln Nebraska USA; ^2^ Water Science Laboratory and Nebraska Water Center University of Nebraska‐Lincoln Lincoln Nebraska USA

**Keywords:** affinity microcolumns, antibiotics, binding studies, humic acid, high‐performance affinity chromatography, micropollutants

## Abstract

High‐performance affinity microcolumns with entrapped humic acid were utilized to investigate interactions between this natural carrier agent and several classes of antibiotics that are common emerging environmental contaminants, or micropollutants. Aldrich humic acid was used as a general model for this type of binding agent. Chromatographic studies under various temperature and mobile phase conditions were used to characterize interactions of the humic acid with the antibiotics sulfadiazine and sulfamethoxazole (sulfonamides), clarithromycin (a macrolide), and lincomycin (a lincosamide). It was determined by this approach that sulfadiazine and sulfamethoxazole had moderate affinities for the humic acid at pH 7.0 and 25°C, with distribution equilibrium constants (*K_D_*) of ∼2–3 × 10^1^ L/kg and global affinities (*nK’_a_*) of ∼0.8–1.0 × 10^3^ M^−1^. Lincomycin and clarithromycin had stronger binding, with *K_D_* and *nK’_a_* values of 3.8–7.5 × 10^2^ L/kg and 1.3–2.6 × 10^4^ M^−1^. All the antibiotics had a negative $\Delta G^{0}$ for this binding, representing spontaneous reactions, and a negative change in enthalpy; however, the change in free energy due to entropy was positive in some cases but negative in others. The binding strength decreased in each case as the ionic strength increased. A change in pH also affected binding, as was consistent with the presence of significant electrostatic interactions from some of the antibiotics. These experiments demonstrated how affinity microcolumns could be employed to study such interactions quickly and with only small amounts of binding agent. The fundamental information obtained through this analytical technique should be valuable in characterizing the transport and activity of these antibiotics in the environment and in adapting this approach to the study of other binding agents and micropollutants that may be found in water.

## 1 Introduction

**FIGURE 1:**
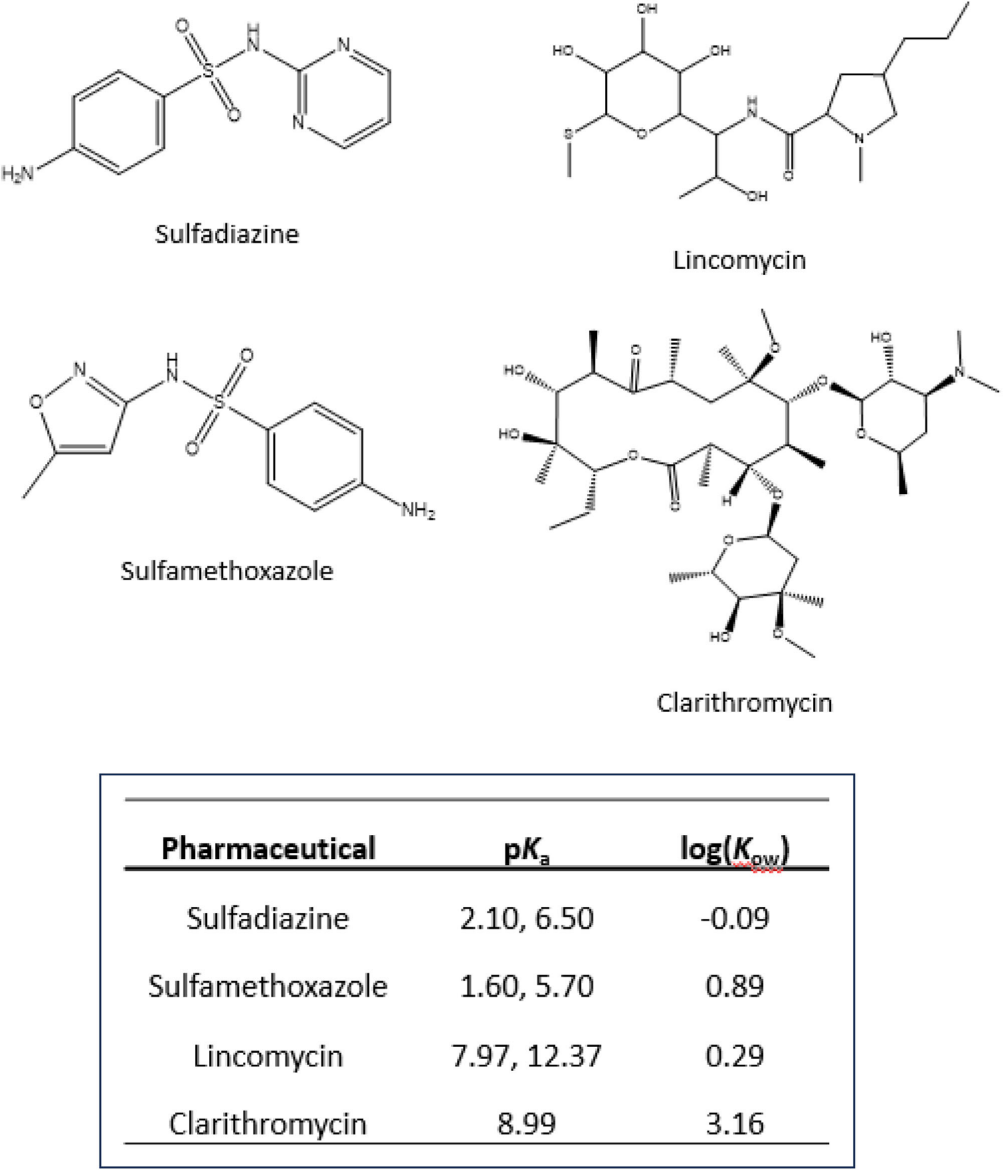
Structures of the antibiotics that were examined in this study. The acid dissociation constants (*K*_a_) for these compounds are also provided in the inset, as listed here in terms of their negative base‐10 logarithm, p*K*_a_. The inset also provides the *n*‐octanol‐water partition coefficients for these compounds (*K*_ow_), as given by the base‐10 logarithm of this value, log(*K*_ow_) [7, 8, 11, 12].

The increased use of various pharmaceuticals globally has led to an unprecedented release of these biologically active compounds into the environment, thereby posing a serious threat to aquatic ecosystems [1, 2, 3, 4, 5, 6]. Traces of pharmaceuticals have been found in surface water, groundwater, and effluents from sewage treatment plants [7, 8]. For instance, sulfadiazine, sulfamethoxazole, lincomycin, and clarithromycin (see Figure 1) are antibiotics that are frequently used in both humans and animals [6, 9, 10, 11, 12]. Readily measured levels of these antibiotics have been detected in multiple environmental compartments [6, 9, 10, 11, 13].

**FIGURE 2:**
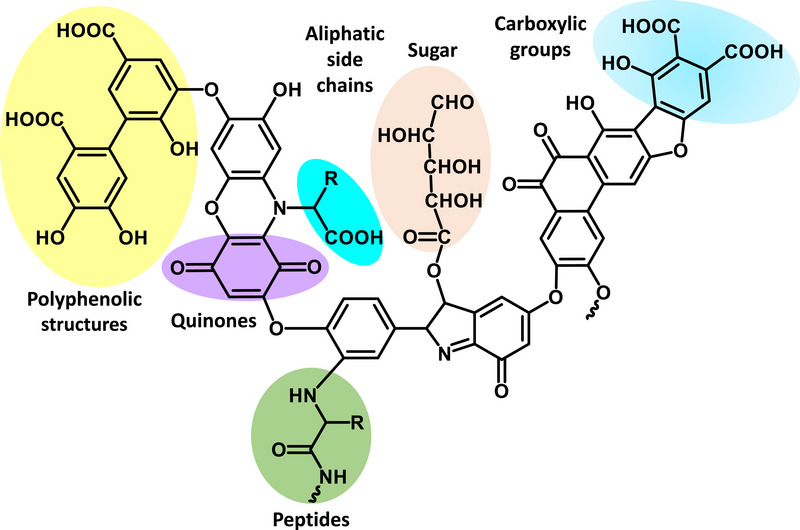
General structure of humic acid, including examples of ionizable acid–base groups that may be present in this structure [16, 18].

Because of their prevalence, it is important to understand how these emerging environmental contaminants, or micropollutants, may interact with natural binding agents that are also present in the environment [1]. Humic acid is a common form of natural organic matter that can bind pharmaceuticals and affect their environmental fate and activity [1, 14]. This substance is produced by the degradation of plant and animal matter and is a major part of dissolved organic matter in water, sediments, and soils [15, 16, 17, 18]. The general structure of humic acid (see Figure 2) consists of a heterogeneous collection of organic polymers (typical mass range, 2–1300 kDa) with carboxyl, enolic, hydroxyl, or phenolic groups and quinones [16, 18]. Peptide or sugar residues may also be present [16, 18].

It is known that humic acid can be an important binding agent for many pharmaceuticals in water and the aquatic environment [19, 20, 21]. However, the complex structure of humic acid makes it difficult to assess the nature of this binding, as this may involve multiple types of functional groups and interactions [22, 23]. In addition, there is limited quantitative information available on the strength of this binding [7, 14, 24]. This is of concern given that the reversible, noncovalent interactions of antibiotics and other pharmaceuticals with humic acid are believed to be important in determining the solubility, transport, and bioavailability of these micropollutants in water and the environment [7, 8, 17, 19, 25].

**FIGURE 3:**
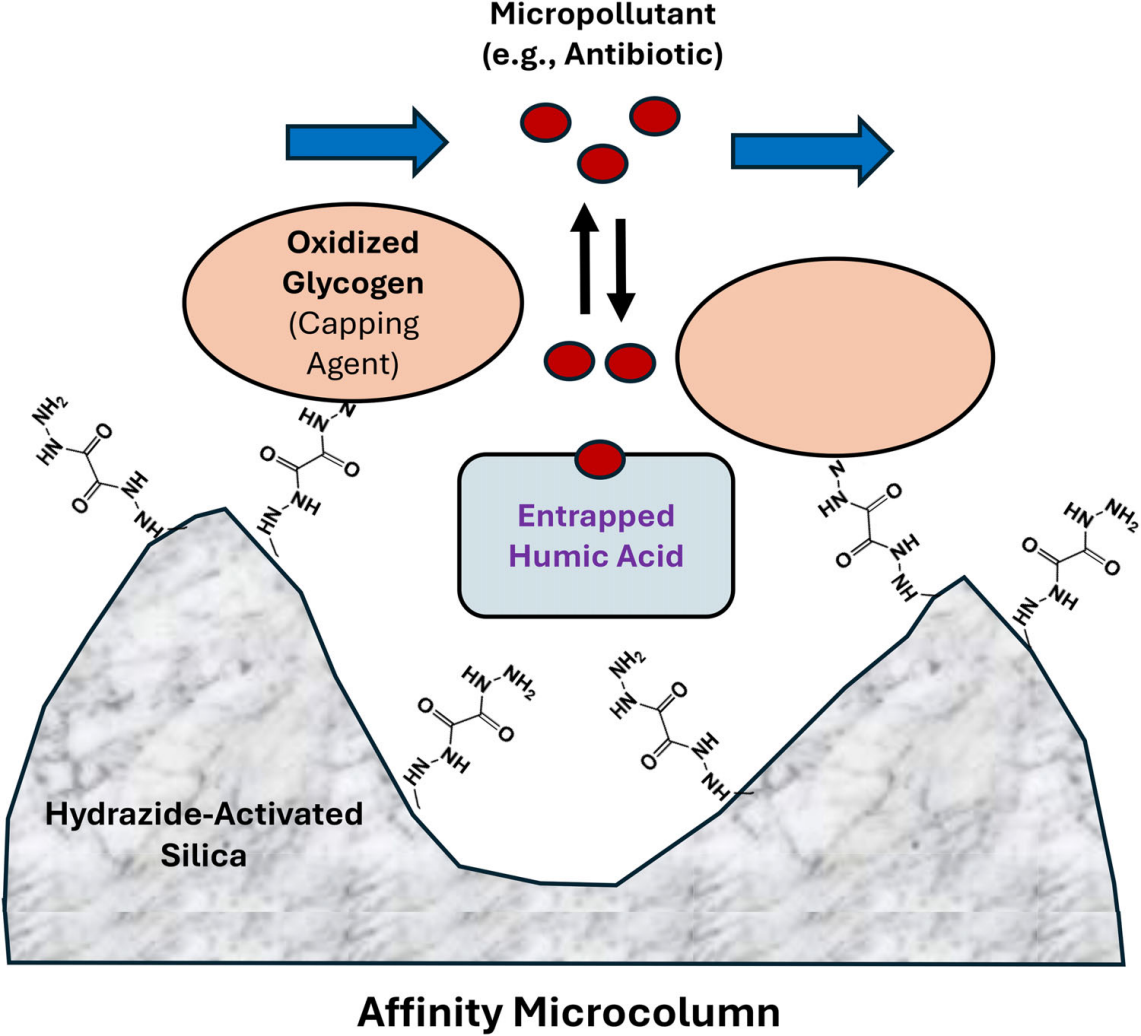
Use of high‐performance affinity microcolumns containing entrapped humic acid for examining the binding of antibiotics or other micropollutants to this agent. The entrapment of humic acid is achieved in this example by placing this agent in a soluble form within the pores or at the surface of HPLC‐grade hydrazide‐activated silica. Mildly oxidized glycogen is also added to this mixture to act as a capping agent, as aldehyde groups on the glycogen become covalently coupled with the hydrazide groups on the support. The final material still allows access of micropollutants to the humic acid, making it suitable for use in chromatographic‐based binding studies.

Methods that have been employed to examine binding and interactions by humic acid have included solid phase extraction, equilibrium dialysis, absorbance or fluorescence spectroscopy, and nuclear magnetic resonance spectroscopy [10, 13, 21, 25, 26, 27]. However, many of these approaches have been limited in their use for this purpose because of their relatively high cost, long separation or equilibration times, and/or need for large sample volumes [10, 13, 21, 26, 27]. An alternative method for studying binding by solutes with humic acid is high‐performance affinity chromatography (HPAC) with noncovalently entrapped samples of humic acid [14, 24]. In this method, the binding agent (e.g., humic acid) is physically entrapped in a soluble form within a porous silica support that can be used in small microliter‐sized columns for HPLC (see Figure 3). Advantages of this approach include its use of only a small amount of humic acid (i.e., a few hundred µg per microcolumn), which can further be used for numerous binding and interaction studies [14, 24, 28, 29, 30, 31]. Other advantages of using HPAC include its ability to be automated and to rapidly acquire precise binding data (i.e., minutes per injection) under various temperature and solution conditions [24, 32].

In this study, HPAC microcolumns containing silica with entrapped humic acid were created and employed to investigate the binding of humic acid with several classes of antibiotics that are found as micropollutants in the environment. These antibiotics included sulfonamides (i.e., sulfadiazine and sulfamethoxazole), lincosamides (lincomycin), and macrolides (clarithromycin) [6, 9, 10, 11]. Affinity microcolumns were made with Aldrich humic acid, an agent often employed as a model for looking at binding by pharmaceuticals and other chemicals with humic acid [14, 21, 24]. After the humic acid content and chromatographic properties of this support were characterized, the overall strength of binding by the entrapped humic acid to each antibiotic of interest was then measured, and the influence of temperature, pH, and salt content or ionic strength of the surrounding solution was evaluated. The results were next compared to the literature and with previous work with humic acid and other types of antibiotics (e.g., tetracycline, norfloxacin, and ciprofloxacin) that have been previously examined by this approach and used as models to develop and validate this method [14, 24]. These measurements gave detailed fundamental data on the extent and nature of these interactions, as now extended to multiple classes of antibiotics, which can be used in the future to model and study their transport, activity, and effects in water and the environment. This work also provided valuable data on the columns and methods that were used in this study, which can be utilized in the future to extend this chromatographic technique and immobilization approach to examine other classes of pharmaceuticals and micropollutants or binding agents that may be found in environmental systems.

## 2 Materials and Methods

### 2.1 Materials

Commercial HPLC‐grade silica (Nucleosil Si‐300, with a pore size of 300 Å and a particle diameter of 7 µm) was purchased from Macherey‐Nagel (Duren, Germany). Sulfadiazine (≥98% pure), sulfamethoxazole (≥98%), lincomycin (≥98%), clarithromycin (≥ 98%), glycogen (bovine liver, type IX, total glucose ≥85%, dry basis), oxalic dihydrazide (98%), and periodic acid (99%) were obtained from Sigma Aldrich (St. Louis, MO, USA). Aldrich humic acid (inorganic residue ∼26.8%, product 53680, lot BCB7247) was also obtained from Sigma Aldrich (Note: see Supporting Material for information on the size or mass distribution of this humic acid preparation, as determined by size‐exclusion chromatography). All other reagents, unless otherwise specified, were of the highest purity grades available. All buffers and aqueous solutions were prepared using purified water from a Milli‐Q system (Dubuque, IA, USA). Amicon ultra centrifugal filters (molecular weight cutoff, 30 kDa; Millipore Sigma, Burlington, MA, USA) were used for the purification of oxidized glycogen. GNWP nylon membrane filters (0.22 µm pore size) were utilized for filtering the buffers (Fischer Scientific, Pittsburgh, PA, USA).

### 2.2 Instrumentation

A ChromTech Prep 24 pump (Apple Valley, MN, USA) was used to pack the microcolumns. Binding studies were performed using an HPLC system that consisted of an LCNet control unit, an autosampler (AS‐2057), a degasser (2080‐54), a pump (PU‐2080), a column oven (CO‐2067), a column selection unit (HV‐2080‐01) to regulate mobile phase and sample flow through the microcolumn, and a detector (UV 2075) from Jasco (Easton, MD, USA). ChromNAV v1.18.04 software from Jasco was used to carry out data acquisition. Fitting and analysis of the chromatographic peaks were performed using PeakFit v4.12 software (Jandel Scientific, San Rafael, CA, USA) with its progressive linear and exponentially modified Gaussian (EMG) functions. The data were also analyzed by using Excel (Microsoft Office 36, Redmond, WA, USA). Thermogravimetric analysis (TGA) was performed on a TGA 550 system (Waters, New Castle, DE, USA) controlled by TRIOS v5.1.46572 software, which was also from Waters.

### 2.3 Preparation of Chromatographic Supports and Microcolumns

The noncovalent immobilization of humic acid within porous HPLC‐grade silica was achieved by using slurry‐based entrapment with split mixing [14]. This approach was adapted from a previous method developed for the entrapment of proteins [29, 30, 31]. Briefly, diol‐bonded silica was first prepared from Nucleosil Si‐300 by placing this support into a pH 5.5, 0.01 M sodium acetate buffer (8.5 mL per g silica), mixing and degassing this solution, and then adding 3‐glycidoxypropyltrimethoxysilane (0.20 mL per g silica). This slurry was then allowed to react at 90°C for 5 h with mixing. After this slurry had been cooled, it was placed in a centrifuge at 8220×*g* for 3 min, and the solution was decanted away from the silica. The silica was washed several times with water and a pH 3.0 solution of dilute sulfuric acid in water, with the centrifugation and decanting steps being repeated at the end of each wash [31]. The silica was next placed in a round‐bottom flask and combined with roughly 150 mL of the pH 3.0 sulfuric acid solution; two glass beads were also added. The flask was attached to a condenser, placed on a heating mantle, and allowed to reflux for 1 h. After refluxing, the diol‐bonded silica was washed several times with water and placed in a drying oven at 60°C [31].

The diol‐bonded silica was next oxidized to obtain aldehyde‐activated silica by using periodic acid, followed by reaction of the aldehyde‐activated support with oxalic dihydrazide to create hydrazide‐activated silica [29, 30, 31]. For this process, the diol‐bonded silica was placed into a reaction container with 1 g periodic acid per g silica and 20 mL of a 90% (v/v) acetic acid solution in water per g silica. This slurry was mixed, degassed, and covered with aluminum foil to protect it from light. The slurry was allowed to react with constant shaking for 2 h at room temperature. The slurry was then placed in a centrifuge at 8220×*g* for 3 min, the solution was decanted away from the silica, and the silica was washed several times with water, followed by further centrifugation and decanting steps. The silica was next resuspended in a pH 5.0, 20 mM sodium acetate buffer containing 150 mM sodium chloride and 1 mg/mL oxalic dihydrazide; this was done using a solution volume sufficient to give a fivefold mol excess of oxalic dihydrazide versus diol groups on the support (e.g., 17.7 mg oxalic dihydrazide for 0.1 g of diol‐bonded Nucleosil Si‐300). The new slurry was mixed and reacted with constant shaking at room temperature for 1–2 h. The silica was next centrifuged and washed several times with pH 7.0, 0.10 M potassium phosphate buffer, using similar conditions to those described for the prior washing and decanting steps. After the final wash and decanting step, the support was placed in a fume hood and combined with pH 8.0 and 0.10 M potassium phosphate buffer containing 28.4 mg sodium borohydride per 0.10 g diol‐bonded Nucleosil Si‐300. This mixture was reacted in a fume hood while being stirred for 90 min at room temperature. The final hydrazide‐activated silica was centrifuged and washed several times with pH 7.0, 0.10 M potassium phosphate buffer, as described for the previous washing steps, and either used immediately for entrapment or stored in pH 7.0, 0.10 M potassium phosphate buffer at 4°C until use (i.e., within 2–4 weeks of support preparation) [31].

A stock solution of humic acid (80 mg/mL) was prepared by stirring 400 mg of Aldrich humic acid in 5.0 mL of 0.10 M potassium phosphate buffer at pH 11.0 for 4 h at room temperature [14, 34, 35]. The pH of the stock solution was then gradually adjusted to 6.0 by adding pH 2.5, 0.10 M potassium phosphate buffer. The resulting humic acid solution (final concentration, ∼14 mg/mL) was added to 70 mg of hydrazide‐activated silica, producing a slurry containing 600 mg humic acid per gram hydrazide‐activated silica. This slurry was then degassed and placed on a wrist‐action shaker for 3.5 h at room temperature. Mildly oxidized glycogen (concentration, 4.25 mg/mL) was prepared by combining 17 mg glycogen in a pH 6.0 buffer containing periodic acid (i.e., ∼135 mg periodic acid dissolved in 4 mL of pH 6.0, 0.10 M potassium phosphate buffer) and allowing this mixture to react for 16 h in the dark at room temperature [14, 36, 37]. These conditions have been shown to give a ∼0.5% oxidation level for glycogen (i.e., when expressed as moles of aldehyde groups produced per mole of glycogen) [30, 38]. A 0.30 mL portion of the oxidized glycogen solution was then added to a 2.95 mL slurry containing the humic acid and hydrazide‐activated silica (i.e., after mixing for 3.5 h). This gave a final mixture containing 18 mg oxidized glycogen per gram silica. This new slurry was then reacted for 18 h at room temperature on a wrist‐action shaker [24, 36, 37]. The unreacted aldehyde groups on the glycogen or on the hydrazide‐activated silica were removed by adding 50 µL of 1 mg/mL oxalic dihydrazide prepared in a pH 6.0, 0.10 M potassium phosphate buffer and allowing the combined reagents to mix and react on a wrist‐action shaker for 2 h at room temperature [37].

A control support was prepared in the same manner as described for the humic acid silica, but with only a pH 6.0 buffer being used in place of the humic acid solution during the immobilization process. In this way, the elution of each antibiotic could be evaluated on both a column with a support containing entrapped humic acid and on a control column with a support prepared in the same way and from an identical starting material but with no soluble humic acid being present (i.e., thus resulting in the same expected surface area and porosity for each support) [14, 31]. Both the humic acid and control supports were downward slurry packed into stainless‐steel microcolumns with lengths of 1.00 cm and an inner diameter (i.d.) of 0.21 cm. A pH 7.4, 0.067 M potassium phosphate buffer was used as the packing solution at 4000 psi (28 MPa). All the microcolumns and supports were stored in the same buffer at 4°C when not in use.

### 2.4 Thermogravimetric Analysis

Thermogravimetric analysis (TGA) was utilized to determine the amount of entrapped humic acid that was present in the supports. This was done in a system operated with nitrogen flow at 20 mL/min to maintain inert conditions during the analysis. The samples were first heated from room temperature to 110°C at 5°C/min. Residual moisture in the sample was removed by holding the sample at this temperature for 20 min. The temperature was next increased from 110°C to 650°C at 20°C/min, with the samples then being held at 650°C for another 10 min [39, 40]. The amount of entrapped humic acid was calculated from these data as described in the Supporting Information Material.

### 2.5 Chromatographic Studies

The effect of temperature on binding by humic acid was evaluated using pH 7.0, 0.10 M potassium phosphate buffer as the mobile phase and temperatures ranging from 10°C to 45°C at a typical flow rate of 0.10 mL/min (total range used in all studies, 0.05–0.50 mL/min) [24]. The initial performance and long‐term stability of the microcolumns were monitored by employing the same mobile phase at 25°C and 0.50 mL/min and by making injections of carbamazepine as a reference compound with known binding properties for Aldrich humic acid [14, 24]. Similar chromatographic conditions were employed at 0.10 mL/min to observe the effects of ionic strength on the retention behavior for each antibiotic in the presence of 0.10–0.40 M NaCl and pH 7.0, 0.10 M potassium phosphate buffer, or to examine this binding when varying the pH of 0.10 M potassium phosphate from 3.0 to 8.0 [24, 27, 41]. The samples were prepared in the respective mobile phases and stored at 4°C when not in use; all antibiotic or solute solutions were used within one week of preparation. The sample concentration for carbamazepine (i.e., a typical pharmaceutical micropollutant used here for initial evaluation of the humic acid microcolumns), sulfadiazine, sulfamethoxazole, and lincomycin was 20 µM, while 10 µM solutions of clarithromycin were employed. These concentrations have been noted to give linear elution conditions for chromatographic‐based binding studies (i.e., retention times and retention factors that are independent of the sample's concentration, as assumed by the equations used in Section 3.2) [14]. Sample injections of 20 µM sodium nitrate were used as a nonretained void marker for the microcolumns and system [14, 24]. All injections were made in replicate (n = 4) using a sample volume of 20 µL. The injected compounds were monitored at the following wavelengths: 286 nm, carbamazepine; 256 nm, sulfadiazine; 266 nm, sulfamethoxazole; 210 nm, lincomycin; 288 nm, clarithromycin; and 205 nm, sodium nitrate [14, 24]. Before sample injections, each microcolumn was equilibrated with the desired mobile phase at 25°C and 0.10 mL/min for 2.5 h.

## 3 Results and Discussion

### 3.1 Characterization of Humic Acid Silica

The measured humic acid content of the chromatographic supports prepared in this study, as determined by TGA, was 20.7 (±4.0) mg Aldrich humic acid per g of silica. This value aligned with those reported previously using similar immobilization conditions [24]. The entrapment of Aldrich humic acid within this type of support has also been confirmed in prior work using attenuated total reflectance FTIR and energy‐dispersive X‐ray spectroscopy, where the spectra and signals obtained by these methods were compared for the humic acid support with a control support that had been prepared in the same manner but with no humic acid added [14]. In addition, analysis based on scanning electron microscopy has shown that no aggregation or cross‐linking occurs for the support during the entrapment process, either upon using oxidized glycogen to cap the hydrazide‐activated silica or upon entrapment of humic acid within this material [14].

The humic acid silica was packed in 1.00 × 0.21 cm i.d. microcolumns, giving a humic acid content of approximately 300 µg per microcolumn. Each of these microcolumns showed good long‐term stability and was typically used over 6 months and 200–300 experiments (e.g., for studies at pH 7.0); this was the equivalent of requiring only 1.1–1.6 µg humic acid per experiment [14, 24]. The ability to reuse the same humic acid preparation in this fashion allowed good precision and reproducibility to be obtained with such microcolumns in binding and thermodynamic studies [24].

The binding activity of the Aldrich humic acid microcolumns was initially tested and then routinely monitored through the injection of carbamazepine. Carbamazepine was used for this purpose because it is known to undergo noncovalent, reversible interactions with this type of humic acid and has known equilibrium constants for this binding [14, 24]. When carbamazepine was injected onto one of these microcolumns at 25°C and pH 7.0 (0.10 M potassium phosphate buffer), the retention times, retention factors, and estimated binding constants were all consistent with values previously reported at the same temperature and pH [24]. The back pressures observed for these microcolumns were also consistent with previous observations based on the same column size and type of support [24]. At 0.10 mL/min, the back pressure for one of these microcolumns was 1.1–1.4 MPa (160–200 psi); at 0.50 mL/min, the back pressure was typically 3.5–5.5 MPa (500–800 psi).

Additional studies with the humic acid and control supports were conducted with injections of low‐ and high‐mass solutes to examine the porosity of these materials (see Supporting Information Materials for details). The void times observed for sodium nitrate, as a low‐mass and nonretained solute, showed no significant difference (e.g., at the 95% confidence level) for columns containing the control support and supports made using the same starting materials but with entrapped humic acid present. This result indicated that the presence of the humic acid did not significantly affect the porosity and accessible pore volume for low‐mass solutes. It was also found that no significant difference, at the 98% confidence level, was present in the void time and accessible pore volume for this low‐mass and nonretained solute when comparing an inert form of the support before it was used in entrapment (i.e., diol‐bonded silica) and the support after it had been taken through the entrapment process. However, when elution for a high‐mass and nonretained solute (i.e., human serum albumin) was examined in the same manner, it was found that this time was ∼24% less than the void time based on the low‐mass and nonretained probe. This difference was expected as a large solute, such as a protein, should have been prevented from reaching the region of the pore that contained the entrapped humic acid.

### 3.2 Chromatographic Measurement of Binding Constants for Antibiotics with Humic Acid

**FIGURE 4:**
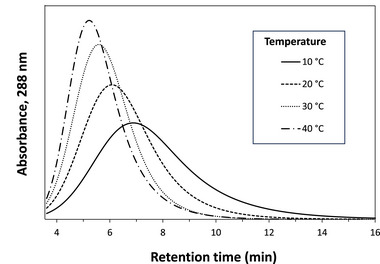
Typical chromatograms and overall retention of peaks at various temperatures for clarithromycin on a 1.00 cm × 0.21 cm i.d. microcolumn containing entrapped Aldrich humic acid and used in the presence of pH 7.0, 0.10 M potassium phosphate buffer, and at a flow rate of 0.10 mL/min.

The microcolumns prepared in this work were next used in chromatographic studies to examine the binding of various antibiotics with Aldrich humic acid. This was done by using the retention observed for each of these antibiotics on the humic acid microcolumns. Examples of chromatograms obtained in these experiments are provided in Figure 4. As shown in these examples, retention data were typically obtained in only a few minutes (e.g., less than 4 min for lincomycin and 10–12 min for clarithromycin at 0.10 mL/min). The differences in the retention factors determined on the humic acid versus control microcolumns were then used to obtain the specific retention factor ($k^{\prime })$ for each antibiotic due to the entrapped humic acid (see Supporting Information Material for retention data).

Several initial observations were made based on these specific retention factors. For instance, these values allowed rapid and quantitative ranking of the overall binding strengths for the various tested antibiotics with the entrapped Aldrich humic acid [14, 24]. At 25°C and in a pH 7.0, 0.10 M potassium phosphate buffer, the order of this binding (from weakest‐to‐strongest) was as follows: sulfamethoxazole, *k’* = 0.20 (±0.05); sulfadiazine, *k’* = 0.28 (±0.05); lincomycin, *k’* = 3.50 (±0.32); and clarithromycin, *k’* = 6.96 (±0.33). The specific retention factors had a precision of ± 4.7%–25% (mean, ± 14%) under these given conditions and similar values at the other temperature, pH values, and ionic strengths examined throughout this report. The consistency of retention values across the tested flow rate range (0.05–0.25 mL/min) confirmed the establishment of a local equilibrium at each peak's central moment [32]. The utilization of specific retention factors corrected for any nonspecific binding by the antibiotics to the microcolumn support when examining the binding by these micropollutants to humic acid [14, 24]. This nonspecific binding accounted for 47% of the total retention measured for sulfadiazine on a humic acid microcolumn, 62% for sulfamethoxazole, 10% for lincomycin, and 56% for clarithromycin in the presence of pH 7.0, 0.10 M potassium phosphate buffer at 25°C.

The specific retention factors were further used to determine binding constants for each antibiotic with the entrapped sample of humic acid. This was done through Equations (1) and (2) [14, 32, 42]:

(1) $$k^{\prime }=\left(n{K^{\prime }}_{a}\right)\frac{m_{L}}{V_{M}},$$

(2) $$k^{\prime }=\frac{\left(K_{D}\right)\;m_{g}}{V_{M}}\;.$$

where, in Equation (1), $K_{a}$ is the association equilibrium constant, as is used when the solute and binding agents have concentrations that are given in units of mol/L [32, 42], and in Equation (2), $K_{D}$ is the distribution equilibrium constant (or partition coefficient) for the same interaction, which is employed when the amount of binding agent is given in units of mass‐per‐volume (e.g., kg/L). This latter type of equilibrium constant (*K_D_*) has a value that is independent of the molar mass of the binding agent, such as may be used in situations where the molar mass of this agent is unknown or not well‐characterized [14]. The term *n* in Equation (1) is the total number of sites (in mol/mol binding agent) involved in the interaction, resulting in a combined term $n{K^{\prime }}_{a}$ that is known as the global affinity [32, 42]. For an agent that has a single type of binding site and where *n* = 1, the term $n{K^{\prime }}_{a}$ in Equation (1), can be replaced by *K_a_* [42]. The term *m_L_* in Equation (1) is the total number of moles of active binding sites in the microcolumn, and *m_g_* in Equation (2), is the mass of the binding agent [14]. In both Equations (1) and (2), *V_M_* is the void volume of the microcolumn [42].

Both $n{K^{\prime }}_{a}$ or $K_{D}$ have been used in prior work to investigate and describe the binding of pharmaceuticals and other solutes with humic acid and related forms of dissolved organic matter [14, 21, 24, 25, 26]. While the affinity constants $n{K^{\prime }}_{a}$ and *K_a_* are often used to describe systems such as drug–protein interactions where the binding agent's molar mass is known [33, 36], the distribution constant $K_{D}$ is particularly well suited for interactions involving agents like humic acid, where the binding agent's molar mass may be unknown or variable. If the average molar mass ($M_{w}$) for such a binding agent can be estimated or is known, conversion between $n{K^{\prime }}_{a}$ (or $K_{a}$) and $K_{D}$ is possible by using the relationship $n{K^{\prime }}_{a}=K_{D}M_{w}$ (or *Ka* =$K_{D}\;M_{w}$ for a system where *n* = 1) [14]. When using either Equations (1) or (2), the specific retention factor that is measured in a chromatographic system for a solute with a binding agent should be proportional to the value of either $n{K^{\prime }}_{a}$ or $K_{D}$ [14, 24].

Table 1 shows the $K_{D}$ and $n{K^{\prime }}_{a}$ values that were determined in this study for the tested antibiotics with Aldrich humic acid at 25°C and pH 7.0 (in 0.10 M potassium phosphate buffer). The equilibrium constants that were acquired at other temperatures are given in the Supporting Information Material. In this process, $K_{D}$ was acquired from $k^{\prime }$ by using Equation (2) and the measured humic acid content of the support (i.e., to provide $\frac{\;m_{g}}{V_{M}}$); $n{K^{\prime }}_{a}$ was then estimated from $K_{D}$ by using the estimated average molar mass for the humic acid (i.e., a value of ∼35 kDa, as used in prior studies with the same type of humic acid and other pharmaceuticals) [14, 24].

**TABLE 1:** Equilibrium constants estimated for the interaction of several common antibiotics with Aldrich humic acid in a pH 7.0, 0.10 M potassium phosphate buffer at 25°C.

Antibiotic	$K_{D}$ (L/kg)^a^	$n{K^{\prime }}_{a}$ (L/mol)^b^
Sulfadiazine	2.98 (±0.75) × 10^1^	1.04 (±0.26) × 10^3^
Sulfamethoxazole	2.16 (±0.39) × 10^1^	7.55 (±1.34) × 10^2^
Lincomycin	3.76 (±0.34) × 10^2^	1.32 (±0.12) × 10^4^
Clarithromycin	7.49 (±0.36) ×10^2^	2.62 (±0.11) × 10^4^

^a^ Each distribution equilibrium constant ($K_{D})$ in this table was calculated using the average $k^{\prime }$ value for an antibiotic across various flow rates (as detailed in the Supporting Information) Materials. This calculation also incorporated the measured humic acid content of the support (20.7 mg/g silica) and the support's known packing density (0.45 mg/mL). The values in parentheses represent ±1 SD, based on four sample injections and as determined through error propagation.

^b^ To estimate the global affinity constant $\left(n{K^{\prime }}_{a}\right)$, $K_{D}$ was multiplied by an average molar mass of 35 000 g/mol for Aldrich humic acid (typical range, 20 000–50 000 g/mol). This was the same molar mass as used previously [14, 24], allowing for a direct comparison of the results from this study to those of earlier work with the same type of humic acid and other types of pharmaceuticals. The use of other estimates for the molar mass of the humic acid will increase or decrease these values for 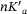 proportionally. Further information on the size distribution of the humic acid used in this study is provided in the Supporting Information Materials.

The results in Table 1 at pH 7.0 and 25°C for Aldrich humic acid showed good agreement with prior studies examining these or similar interactions with other types of humic acids and by other techniques. For instance, the $n{K^{\prime }}_{a}$ of 1.04 (±0.26) × 10^3^ M^−1^ shown for sulfadiazine in Table 1 was comparable to the result of ∼3 × 10^3^ M^−1^ that has been reported for this antibiotic with purified humic acid (unspecified source) at pH 7.0 and 25°C when using fluorescence quenching [26]. The $K_{D}$ of 2.16 (±0.39) × 10^1^ L/kg in Table 1 for sulfamethoxazole was in the same range as the reported value of 4.6×10^1^ L/kg at 25°C and an unspecified pH for this antibiotic with a commercial preparation of humic acid [10]. The $K_{D}$ for clarithromycin of 7.49 (±0.36) × 10^2^ L/kg in Table 1 was in the same range as a value of 19 (±4) × 10^2^ L/kg that was obtained by equilibrium dialysis at pH 6.5 for clarithromycin with Elliott soil humic acid [25]. The $K_{D}$ of 3.76 (±0.34) × 10^2^ L/kg that was measured for lincomycin binding with Aldrich humic acid at pH 7.0 was the same magnitude as a $K_{D}$ of 1.9 × 10^3^ L/kg that was found for this antibiotic at pH 9.0 and room temperature with Aldrich humic acid when using solid‐phase extraction [21].

A comparison was next made between the binding constants in Table 1 and the relative polarities of the antibiotics. This was done by using the values of the base‐10 logarithm of the *n*‐octanol‐water partition coefficient, or log(*K*_ow_), as a general measure of polarity for each antibiotic (see Figure 1) [7, 8, 11, 12]. This study found that the order of the binding strength for the tested antibiotics to Aldrich humic acid at 25°C and pH 7.0 did not follow their log(*K*_ow_) values. For instance, the binding strength order was sulfadiazine ≈ sulfamethoxazole < lincomycin ≈ clarithromycin, while the polarity order (from most polar to least polar, based on log (*K*_ow_)) was sulfadiazine < lincomycin < sulfamethoxazole < clarithromycin. This observation aligns with previous research indicating that the environmental mobility of pharmaceuticals is not solely determined by their polarities [1, 24]. Instead, the presence of multiple polar, nonpolar, and ionizable functional groups in these pharmaceuticals suggests that a variety of potential interactions can occur for these substances with binding agents like humic acid [1]. These interactions were further investigated in the following sections by using the humic acid microcolumns.

### 3.3 Effect of Temperature on Binding by Humic Acid with Antibiotics

The effect of temperature on the retention and binding strength of the selected antibiotics with the Aldrich humic acid microcolumns was next considered. The results are tabulated in Table 2 and the Supporting Information Material. Typical chromatograms for clarithromycin in these temperature studies are provided in Figure 4, with the other antibiotics showing similar behavior. In all cases, and in the presence of pH 7.0, 0.10 M potassium phosphate buffer, the observed retention and binding strength (i.e., based on the relationship between these parameters in Equations (1) and (2)) decreased as the temperature was raised from 10°C to 45°C. For sulfadiazine and sulfamethoxazole, the binding strength with Aldrich humic acid declined by up to 68%–70% over 10–45°C. Lincomycin and clarithromycin decreased in their binding strengths by 13% and 51%, respectively, under the same conditions.

**TABLE 2:** The specific retention factors of several common antibiotics determined using an Aldrich humic acid microcolumn at various temperatures and in pH 7.0, 0.10 M potassium phosphate buffer^a^.

Temperature (°C)	${k^{\prime }}_{\mathrm{Sulfadiazine}}$	${k^{\prime }}_{\mathrm{Sulfamethoxazole}}$	${k^{\prime }}_{\mathrm{Lincomycin}}$	${k^{\prime }}_{\mathrm{Clarithromycin}}$
10	0.47 (±0.02)	0.33 (±0.03)	3.30 (±0.08)	8.52 (±0.24)
20	0.32 (±0.03)	0.22 (±0.03)	3.08 (±0.05)	6.86 (±0.20)
25	0.28 (±0.02)	0.20 (±0.02)	3.05 (±0.06)	6.21 (±0.24)
30	0.23 (±0.03)	0.17 (±0.03)	3.03 (±0.15)	4.97 (±0.14)
37	0.18 (±0.02)	0.13 (±0.03)	3.01 (±0.04)	4.41 (±0.13)
40	0.18 (±0.03)	0.12 (±0.02)	2.91 (±0.09)	4.30 (±0.11)
45	0.15 (±0.03)	0.10 (±0.02)	2.86 (±0.04)	4.15 (±0.10)

^a^ The specific retention factors were based on data collected at 0.10 mL/min using humic acid and control microcolumns with sizes of 1.00 cm × 0.21 cm i.d. Other conditions are given in the text. The values in parentheses represent ±1 SD for four sample injections.

The values of $n{K^{\prime }}_{a}$ that were obtained at these temperatures were also used to estimate the changes in free energy during the binding of the antibiotics with Aldrich humic acid. As an example, the change in the standard Gibbs free energy $\left(\Delta G^{0}\right)$ was calculated by using Equation (3) [24, 26]:

(3) $$\Delta G^{0}=-\mathrm{RT}\;\mathrm{In}\left(n{K^{\prime }}_{a}\right),$$

where *T* is the absolute temperature, and *R* is the ideal gas law constant. The $\Delta G^{0}$ values that were acquired at 25°C by using Equation (3) are listed in Table 3. The negative $\Delta G^{0}$ values show that these reactions were spontaneous [13]. $\Delta G^{0}$ was −16.4 to −17.2 kJ mol^−^^1^ for sulfadiazine and sulfamethoxazole and −23.2 and −24.9 kJ mol^−^^1^ for lincomycin and clarithromycin at 25°C. The more negative $\Delta G^{0}$ values for these last two antibiotics corresponded to their higher affinities than sulfadiazine or sulfamethoxazole for Aldrich humic acid. The general range of all these $\Delta G^{0}$ values was consistent with prior estimates made at 25°C for some of the same drugs with other types of purified or commercial preparations of humic acid (e.g., sulfadiazine at pH 8.0 or sulfamethoxazole at an unspecified pH) [10, 26].

**TABLE 3:** The thermodynamic parameters of several common antibiotics in the presence of Aldrich humic acid and pH 7.0, 0.10 M potassium phosphate buffer^a^.

Antibiotic	$\Delta G^{0}$ (kJ mol^−^^1^) at 25°C	$\Delta H^{0}$ (kJ mol^−^^1^)	$\Delta S^{0}$ (J mol^−^^1^K^−^^1^)
Sulfadiazine	−17.2 (±0.2)	−23.9 (±0.9)	−22.5 (±2.9)
Sulfamethoxazole	−16.4 (±0.3)	−25.0 (±0.6)	−28.9 (±2.1)
Lincomycin	−23.2 (±0.1)	−2.7 (±0.3)	68.9 (±1.1)
Clarithromycin	−24.9 (±0.1)	−16.6 (±1.3)	27.5 (±4.1)

^a^ These results are based on data collected at 0.10 mL/min using 1.00 cm × 0.21 cm humic acid and control microcolumns. The values in parentheses represent a range of ±1 SD based on four injections and error propagation. Values for $\Delta G^{0}$ were calculated using Equation (3), and values of $\Delta H^{0}$ and $\Delta S^{0}$ were obtained from plots generated according to Equation (5).

It was further possible to calculate the changes in the standard enthalpy $\left(\Delta H^{0}\right)$ and standard entropy $\left(\Delta S^{0}\right)$ of the interactions for each antibiotic with Aldrich humic acid. For example, van't Hoff equation can be used to show the relationship of $\Delta G^{0}$ with $\Delta H^{0}$ and $\Delta S^{0}$ (Equation 4) [13, 26, 41]:

(4) $$\Delta G^{0}=\Delta H^{0}-T\Delta S^{0}$$

Moreover, Equations (3) and (4) can be put together to generate a similar relationship that is based on $n{K^{\prime }}_{a}$ instead of $\Delta G^{0}$ [43, 44]:

(5) $$\mathrm{In}\left(n{K^{\prime }}_{a}\right)=-\frac{\Delta H^{0}}{RT}+\frac{\Delta S^{0}}{R}.$$

Equation (5) suggests a plot of ln $\left(n{K^{\prime }}_{a}\right)$ versus 1/T for simple solute‐ligand binding should give a linear relationship with a slope of $-\frac{\Delta H^{0}}{R}$ and an intercept equal to $\frac{\Delta S^{0}}{R}$, which can then be used to obtain $\Delta H^{0}$ and $\Delta S^{0}$ [43].

**FIGURE 5:**
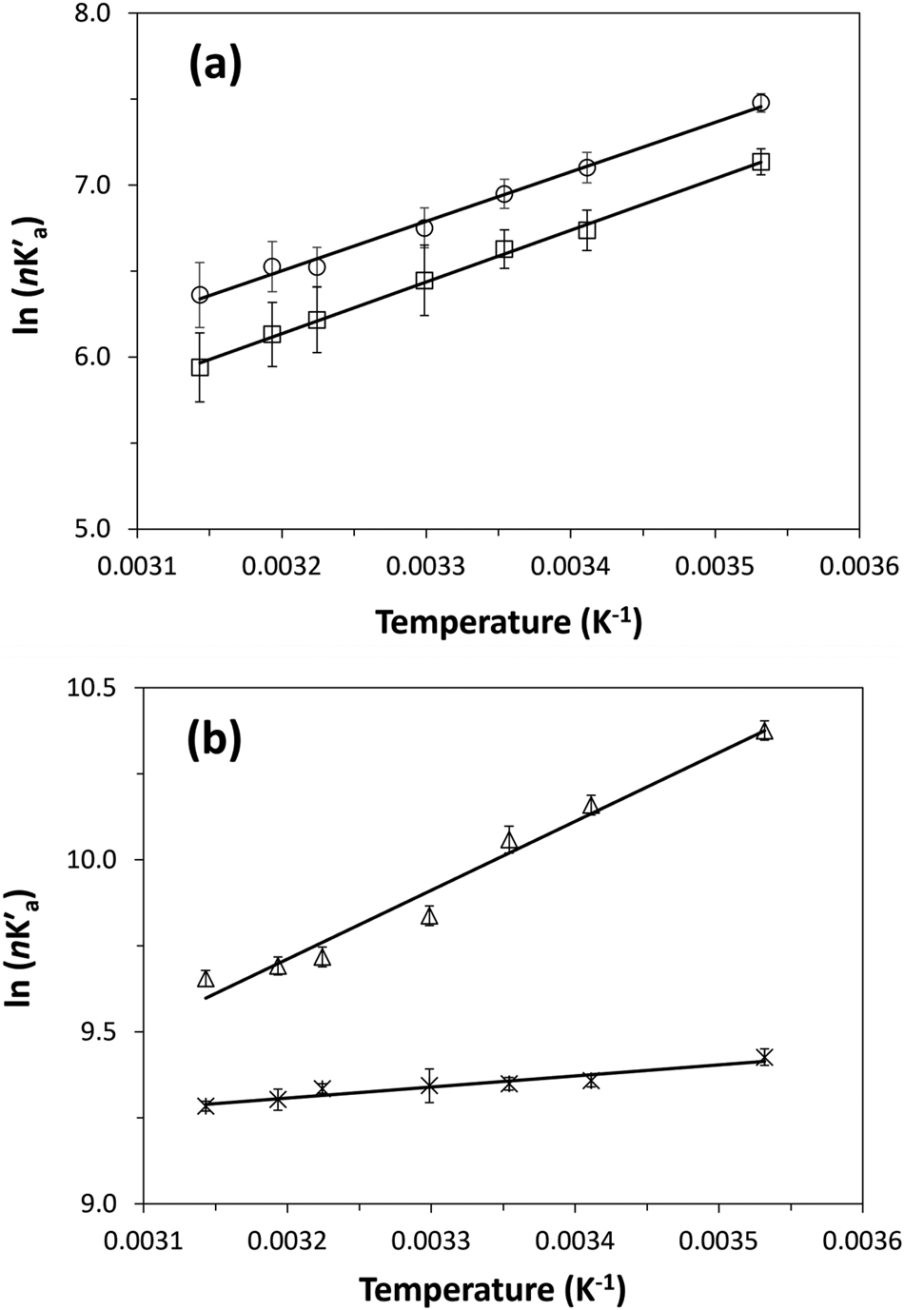
Plots prepared according to Equation (5) for zonal elution studies with sulfadiazine (○), sulfamethoxazole (□), lincomycin (*), and clarithromycin (∆), on a 1.00 cm × 0.21 cm i.d. microcolumn containing Aldrich humic acid and used at 0.10 mL/min and temperatures of 10°C to 45°C. Other experimental conditions are given in the text. The equations for the best‐fit lines were as follows: *y* = [2.8 (±0.1) × 10^3^] *x*—[2.7 (±0.4)], with a correlation coefficient of 0.9965 for sulfadiazine; *y* = [3.0 (±0.1) × 10^3^] *x*—[3.5 (±0.2)], with a correlation coefficient of 0.9984 for sulfamethoxazole; *y* = [3.2 (±0.4) × 10^2^] *x* + [8.3 (±0.1)], with a correlation coefficient of 0.9607 for lincomycin; and *y* = [2.0 (±0.2) × 10^3^] *x* + [3.3 (±0.5)], with a correlation coefficient of 0.9861 for clarithromycin (*n* = 7 temperatures for all plots). The error bars represent a range of ± 1 SD for four injections. The relative precisions of the *y*‐values ranged from ±0.1% to 3.4%.

Figure 5 presents plots created by using Equation (5) and retention data for each antibiotic on an Aldrich humic acid microcolumn. The strong linearity observed in these plots across temperatures of 10–45°C (correlation coefficients ranging from 0.9607 to 0.9984, *n* = 7) suggests that the interaction between these antibiotics and Aldrich humic acid follows a simple reversible binding model [43, 45]. Similar behavior has been reported for the binding by other pharmaceuticals and antibiotics with Aldrich humic acid [24].

The values obtained for $\Delta H^{0}$ and $\Delta S^{0}$ are listed in Table 3. $\Delta H^{0}$ ranged from −2.7 to −25.0 kJ mol^−^^1^ for all the tested antibiotics with Aldrich humic acid, indicating the overall bond formation versus bond breaking during these interactions was energetically favored. For sulfadiazine and sulfamethoxazole, $\Delta S^{0}$ was negative (i.e., −22.5 and −28.9 J mol^−^^1^K^−^^1^); this resulted in a positive value for $-T\Delta S^{0}$ value at 25°C, indicating there was a decrease in overall entropy during the binding of these antibiotics to Aldrich humic acid at this temperature. However, for lincomycin and clarithromycin $\Delta S^{0}$ was positive (68.9 and 27.5 J mol^−^^1^K^−^^1^) and $-T\Delta S^{0}$ was negative at 25°C. This created a situation at this temperature in which there was a net increase in overall entropy during the binding of humic acid with lincomycin or clarithromycin, which can occur due to disruption of solvent structure during the formation of a solute‐ligand complex [43]. The combination of negative $\Delta H^{0}$ and $\Delta S^{0}$ values for sulfadiazine and sulfamethoxazole were consistent with prior results for the interactions by these antibiotics with a commercial preparation of humic acid and activated carbon [13, 46]. The negative $\Delta H^{0}$ and positive $\Delta S^{0}$ values for lincomycin and clarithromycin agreed with behavior noted for other pharmaceuticals during their binding to humic acid, including Aldrich humic acid [10, 24].

### 3.4 Effect of pH on Interactions of Humic Acid with Antibiotics

The mobile phase pH was modified to further investigate the interactions between the tested antibiotics and Aldrich humic acid. The pH is known to affect both the charge and structure of humic acid [27, 47, 48]. At low pH values, protonation of carboxylic acid groups (p*K*_a_ ∼ 4.5) on humic acid promotes aggregation of this substance via intra‐intermolecular hydrogen bonding [27]. This change, in turn, may alter the extent to which hydrophobic versus polar interactions play a role in the binding of substances to humic acid [47, 49].

The effect of pH on the Aldrich humic acid ionization has previously been examined by measuring its zeta potential at 25°C and in 0.10 M potassium phosphate solutions at pH 2.0–8.0 [24]. A negative zeta potential (from −15.5 to −35.9 mV) was present for the humic acid over this pH range, as was expected due to dissociation and deprotonation of acidic functionalities on this binding agent, such as carboxylate (p*K*_a_ range, 3.1–4.5) and phenolic (p*K*_a_ range, 6.0–10) groups [24, 50]. In addition, the pH‐dependent dissociation of these acidic groups can disrupt intramolecular hydrogen bonds, which has been proposed to lead to an expansion of humic acid and increased exposure of its solute‐binding sites [51].

**FIGURE 6:**
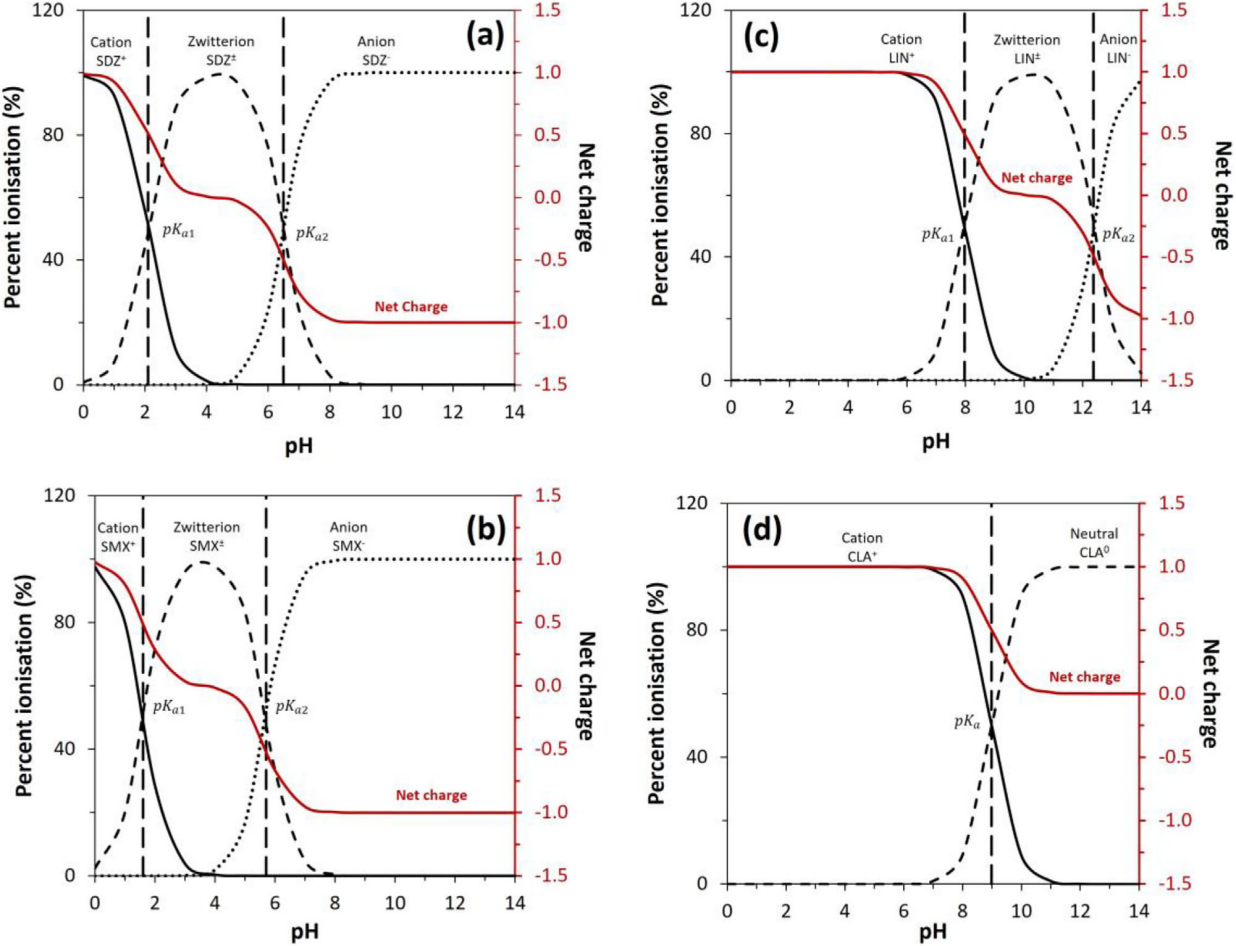
The acid–base forms and their fractions (%) that are present as a function of pH for (A) sulfadiazine (SDZ), (B) sulfamethoxazole (SMX), (C) lincomycin (LIN), and (D) clarithromycin (CLA). The net charge as a function of pH for each antibiotic is also provided. These results were calculated as described in the Supporting Information Material.

An alteration in pH can also change the types of acid–base forms that are present for an antibiotic. This can be examined by making plots of the relative fractions of the antibiotic that are present in these forms as a function of pH and using the known p*K*_a_ values for this compound, as listed in Figure 1 [7, 8, 11, 12]. The net charge of each antibiotic as a function of pH can also be calculated based on these results [52]. Plots showing how a change in pH affects both the distribution of the acid–base forms of these antibiotics and their net charges are provided in Figure 6.

**FIGURE 7:**
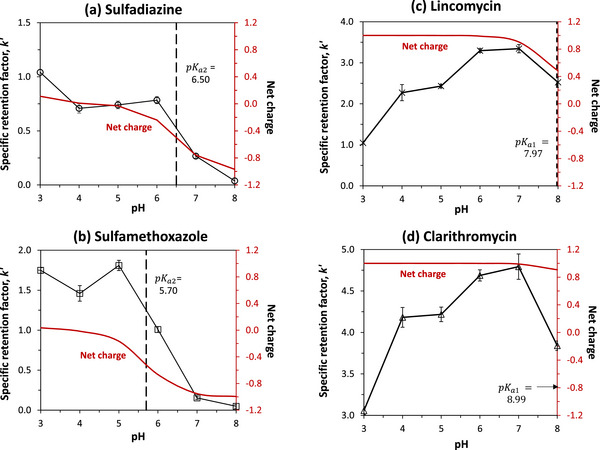
The effect of pH on the specific retention factors for the binding of entrapped Aldrich humic acid with (A) sulfadiazine (○), (B) sulfamethoxazole (□), (C) lincomycin (*), and (D) clarithromycin (∆). These results were obtained by adjusting the pH from 3.0 to 8.0 in 0.10 M potassium phosphate solutions that were used as the mobile phase and by injecting the antibiotics at 25°C onto 1.00 cm × 0.21 cm i.d. microcolumns at 0.10 mL/min. The error bars represent a range of ± 1 S.D. (*n* = 4 injections). The vertical dashed lines represent the p*K*_a_ values of the antibiotics over the given pH range. The net charge for each antibiotic as a function of pH is also provided for reference (see Figure 6 and Supporting Information Material for further details).

The net effect of a change in pH on the binding of these antibiotics to Aldrich humic acid was investigated by measuring the specific retention factors for these solutes on the humic acid microcolumns while varying the mobile phase pH. Figure 7 gives a summary of the results, and the measured values for $k^{\prime }$ are provided in the Supporting Information Material. The p*K*_a_ values present over the pH range of 3.0–8.0 are also provided for reference, as well as the net charges of the antibiotics under these conditions. The given pH range was chosen for this study because it represented conditions over which the silica support in the microcolumn was known to be stable, and it covered pH values seen in most environmental water samples [53].

For sulfadiazine and sulfamethoxazole, the specific retention factors in Figure 7A,B showed a significant change as the pH was varied. The $k^{\prime }$ for sulfadiazine decreased slightly (25%–32%) from pH 3.0 to pH 4.0–6.0 and then showed a further total decrease of about 74% or 96% in reaching pH 7.0 or 8.0. Similar overall behavior was seen for sulfamethoxazole, but with variations of only 3%–16% between pH 3.0 and 5.0, followed by a decrease of 42% at pH 6.0 and 91%–97% at pH 7.0–8.0. In both cases, the pH over which these antibiotics showed their strongest binding was in the region between their first and second p*K*_a_ values, and where both compounds were mainly present as neutral zwitterions. Examples of forces that may have contributed to binding under these conditions included nonpolar interactions, dipole interactions, and hydrogen bonding. However, electrostatic interactions may have also been present, such as between the positively‐charged regions on the zwitterions and humic acid's negatively‐charged residues. The presence of some electrostatic effects would explain why there was a decrease in affinity for these compounds as the pH approached and exceeded their second p*K*_a_, or conditions in which a singly‐charged anion became the dominant acid–base form for these antibiotics. This change in the dominant form would have led to electrostatic repulsion between the antibiotics and humic acid. These general observations are in agreement with prior work examining binding using these sulfonamide‐class antibiotics with soils or humic acid [9, 54].

Different behavior was seen in Figure 7C,D for lincomycin and clarithromycin. Both these compounds had a net positive charge from pH 3.0 to 8.0, which decreased slightly above pH 7.0. The specific retention factors for these antibiotics increased by 1.6‐ to 3.2‐fold in going from pH 3.0 to 7.0 and then decreased by 19%–32% between pH 7.0 and 8.0. Both drugs were mainly present as singly‐charged cations over this pH range, with the upper end of pH 8.0 being at or below their first p*K*_a_ [8, 55]. This situation should have led to an electrostatic attraction between the cations for these drugs and negative charges on humic acid at pH 7.0 or lower [11]. In addition, this attraction should have increased with pH, and as the negative zeta potential and charge on the humic acid continued to increase [24]. Above pH 7.0, some of these antibiotics were also present as neutral zwitterions. This would have led to a small decrease in electrostatic attraction and in overall binding strength, as observed in Figure 7.

### 3.5 Effect of Ionic Strength on Binding by Humic Acid with Antibiotics

To further examine binding mechanisms for the tested antibiotics with Aldrich humic acid, the ionic strength of the mobile phase was altered by adding NaCl. This fully dissociated and inert salt was added to the mobile phases at concentrations ranging from 0.00 to 0.40 M. This resulted in overall ionic strengths for these solutions in a pH 7.0, 0.10 M potassium phosphate buffer of up to almost 0.60 M when ions from the potassium phosphate buffer were also considered.

**FIGURE 8:**
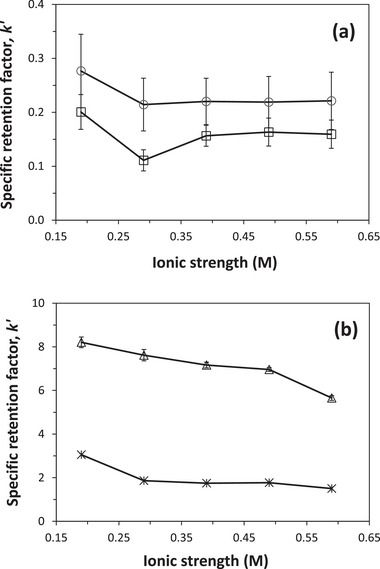
Effect of ionic strength on binding by (A) sulfadiazine (○), sulfamethoxazole (□), (B) lincomycin (*), and clarithromycin (∆) to a 1.00 cm × 0.21 cm i.d. microcolumn containing Aldrich humic acid. The error bars represent a range of ± 1 S.D (*n* = 4 injections) and in some cases are of similar size to the symbols used in these plots (e.g., for lincomycin). These results were obtained in the presence of pH 7.0, 0.10 M potassium phosphate buffer containing 0.10, 0.20, 0.30, or 0.40 M NaCl and by injecting the samples at 25°C and 0.10 mL/min.

Figure 8 shows how the overall binding and specific retention factors on a humic acid microcolumn were affected for each antibiotic as the ionic strength was varied at pH 7.0 and 25°C (see data provided in Supporting Information Material). In general, the binding strength decreased for these interactions as the ionic strength was increased. This decrease was as high as 23% to 45% for sulfadiazine or sulfamethoxazole and 31% or 51% for clarithromycin or lincomycin. The observed changes in antibiotic binding with increasing ionic strength were likely due to a combination of factors: the shielding of dipole and electrostatic interactions between the antibiotics and humic acid [56, 57] and alterations in the electrostatic interactions within humic acid (i.e., as may affect humic acid's conformation and the accessibility of its binding sites) [9, 27].

## 4 Concluding Remarks

In this study, high‐performance affinity microcolumns were used to study the binding of entrapped Aldrich humic acid to several common antibiotics that occur as micropollutants in the environment. These antibiotics included sulfadiazine and sulfamethoxazole as representative sulfonamide‐class antibiotics, lincomycin as a lincosamide‐class antibiotic, and clarithromycin as a macrolide antibiotic. The measured retention of these antibiotics was used to rank their overall binding strength to the humic acid under various conditions and to estimate their equilibrium constants for this binding. It was possible with this approach to often acquire binding data in minutes and by using only small amounts of humic acid (i.e., around 300 µg per microcolumn, which could then be used repeatedly over 200–300 experiments). This method further made it possible to quickly compare the binding of humic acid with multiple classes of antibiotics that may be found as micropollutants in the environment.

At pH 7.0 and 25°C, sulfadiazine and sulfamethoxazole had similar values for their equilibrium constants $K_{D}$ and $n{K^{\prime }}_{a}$ of ∼2–3 × 10^1^ L/kg and ∼0.8–1.0 × 10^3^ M^−1^, respectively. Lincomycin and clarithromycin had 13‐ to 35‐fold stronger binding, with values for $K_{D}$ and $n{K^{\prime }}_{a}$ of around 3.8–7.5 × 10^2^ L/kg and 1.3–2.6 × 10^4^ M^−1^. All these affinities were comparable to or much lower than those measured previously by the same approach and with Aldrich humic acid for two other types of antibiotics: tetracycline ($K_{D}$, 0.84 × 10^3^ L/kg) and fluroquinolones ($K_{D}$, ∼4.8–5.6 × 10^4^ L/kg) [24]. These results agreed with a previous general observation that tetracyclines and fluoroquinolones tend to show stronger binding to humic acid than antibiotics that are macrolides or sulfonamides [58]. In addition, it was noted that the general order of binding strengths for the tested antibiotics with humic acid did not follow the order of their overall polarities, as has also been noted for tetracyclines and fluoroquinolines [24]. This last observation suggested that forces other than nonpolar interactions, such as electrostatic effects, hydrogen bonding, or dipole interactions, were also important for this binding.

The humic acid microcolumns were further utilized to examine the effects of changing temperature, pH, and ionic strength on this binding. All the antibiotics had negative $\Delta G^{0}$ values for this binding, representing spontaneous reactions. For example, $\Delta G^{0}$ at 25°C was between −16.4 and −17.2 kJ mol^−^^1^ for sulfadiazine and sulfamethoxazole, and −23.2 to −25.0 kJ mol^−^^1^ for lincomycin and clarithromycin. For each of these interactions, there was a negative change in enthalpy, as would occur when a net gain in energy was present due to bond formation versus bond breaking. The change in free energy due to entropy was positive in some cases (i.e., for sulfadiazine and sulfamethoxazole) but negative in others (i.e., for lincomycin and clarithromycin).

The pH and ionic strength dependence measured for this retention agreed with the general observations of the thermodynamic studies. An increase in ionic strength for each of the antibiotics resulted in weaker binding, as has also been seen for tetracycline and fluoroquinolones [24]. A change in pH affected binding by all these antibiotics with humic acid, as could be linked to the changes in charge and electrostatic interactions, as well as possible changes in the structure of humic acid and the accessibility of its binding regions. The strongest binding for sulfadiazine and sulfamethoxazole was seen at a pH of 5–6 or lower and when these compounds were mainly present as neutral zwitterions; this binding strength then decreased at higher pH values as the antibiotics were converted into their anionic form and electrostatic repulsion began to occur with humic acid. For lincomycin and clarithromycin, the strongest binding was observed around pH 7, and when these antibiotics were mainly present as singly‐charged cations that could interact with humic acid through electrostatic attraction [11].

The results of this work are important because there is still a limited understanding of how antibiotics and other micropollutants bind with humic acid, which may affect their transport and bioavailability. This study showed how HPAC can efficiently screen and rank the binding by various classes of antibiotics with humic acid and be used to investigate plus compare the mechanisms behind the interactions. The data provided by this study can further be used to adapt this same entrapment method and chromatographic approach to study binding by other micropollutants with humic acid or alternative agents. This data, in turn, can be used in the future to help predict and model how this binding affects the transport, bioavailability, and potential risks of antibiotics and other pharmaceutical contaminants in the environment.

## Author Contributions

**Sadia Sharmeen**: investigation, formal analysis, validation, methodology, writing – original draft. **Isaac Kyei**: validation, methodology, investigation. **Saumen Poddar**: validation, methodology. **Sazia Iftekhar**: methodology, investigation. **BK Sajeeb**: methodology, investigation, writing – review and editing. **Lillian M. Graham**: writing – review and editing. **Daniel D. Snow**: writing – review and editing, visualization, funding acquisition, formal analysis. **David S. Hage**: conceptualization, funding acquisition, formal analysis, visualization, supervision, project administration, writing – review and editing.

## Conflicts of Interest

The authors declare no conflicts of interest.

## Supporting information




**Supporting File**: jssc70345‐sup‐0001‐SupMat.pdf.
