# Supplementary material for: Chromatographic‐Based Binding and Thermodynamic Studies of Antibiotic Micropollutants with Humic Acid Using Affinity Microcolumns
Source: J Sep Sci. 2026 Jan 13;49(1):e70345. doi: 10.1002/jssc.70345 (PMC12797113; doi:10.1002/jssc.70345)
Supplement: Supplementary file 1 — Supporting File: jssc70345‐sup‐0001‐SupMat.pdf. [file JSSC-49-e70345-s001.pdf]

Supplementary Material

Chromatographic-Based Binding and Thermodynamic Studies of Antibiotic Micropollutants with Humic Acid using Affinity Microcolumns

Sadia Sharmeen[1], Isaac Kyei[1], Saumen Poddar[1], Sazia Iftekhar[1], BK Sajeeb[1], Lillian M. Graham[1], Daniel D. Snow[2] and David S. Hage*[1]

[1]Department of Chemistry, University of Nebraska-Lincoln, Lincoln, NE

[2]Water Science Laboratory and Nebraska Water Center, University of Nebraska-Lincoln, Lincoln, NE

*Author for Correspondence: Chemistry Department, University of Nebraska-Lincoln, Lincoln, NE 68588-0304, USA. Phone: 402-472-2744; FAX: 402-472-9402; Email: dhage1@unl.edu

Table of Contents

Topic	Page
Determination of entrapped humic acid by thermogravimetric analysis	3
Retention measurements for antibiotics on humic acid and control microcolumns	3
Determination of fractions of acid-base forms and net charges of antibiotics vs pH	12
Characterization of humic acid by size-exclusion chromatography	15
Characterization of support porosity for entrapment-based immobilization	21

Determination of entrapped humic acid by thermogravimetric analysis

The amount of entrapped humic acid (F_{HA}, in *w/w*) was determined using thermogravimetric analysis. This was done by measuring the relative mass change of the humic acid support compared to a control support as each was heated from 110 °C to 650 °C. The value of F_{HA} was calculated as shown below,

$$F_{HA} = \frac{\%W_{h110} - \%W_{h650}}{\%W_{h110}} - \frac{\%W_{c110} - \%W_{c650}}{\%W_{c110}} \quad \text{(S1)}$$

where $\%W_{h110}$ and $\%W_{h650}$ represent the percentage of initial weights for the humic acid silica at 110 °C versus 650 °C, while $\%W_{c110}$ and $\%W_{c650}$ represent the corresponding percentages and relative weights for the control silica. An additional correction was applied to account for the 26.8% inorganic matter present in the humic acid, as indicated by the supplier [1S]. This correction involved multiplying the F_{HA} value from eq. (S1) by (1 – 0.268), or 0.732, to obtain the corrected, F_{HA_o} value. This result was then multiplied by 1000 to provide the humic acid content in parts-per-thousand (i.e., mg humic acid per g silica) [1S,2S].

Retention measurements for antibiotics on humic acid and control microcolumns

The retention times for the antibiotics were measured using zonal elution under conditions that represented linear elution (i.e., sufficiently low sample concentrations ensuring retention that was not significantly dependent on solute concentration) [1S-4S]. A solute's retention factor (*k*) was determined from the chromatographic data as shown below,

$$k = \frac{t_R - t_M}{t_M - t_0} \quad \text{(S2)}$$

where, t_R is the retention time of the antibiotic or solute, t_M is the column void time, and t_0 is the void time of the chromatographic system with no column present [1S-4S]. The specific retention factor (k') resulting from solute binding to humic acid was then calculated as the difference

between the total retention factors observed on an Aldrich humic acid microcolumn (k_{Total}) and a control column ($k_{Control}$) [1S-4S], as shown in eq. (S3).

$$k' = k_{Total} - k_{Control} \tag{S3}$$

Table 1S compares the specific retention factors obtained for the tested antibiotics at 25 °C and in pH 7.0, 0.10 M potassium phosphate buffer with Aldrich humic acid at several flow rates. Table 2S shows retention data for the same antibiotics at various temperatures and in pH 7.0, 0.10 M potassium phosphate buffer. Table 3S summarizes the distribution equilibrium constants (K_D) and global affinity constants (nK'_a) calculated for these antibiotics using Aldrich humic acid at pH 7.0 in 0.10 M potassium phosphate buffer and various temperatures. Table 4S shows retention data for the same antibiotics and microcolumns at 25 °C and with 0.10 M solutions of potassium phosphate that were prepared at various pH values. Table 5S gives the retention data that were obtained for these antibiotics on the same types of columns in pH 7.0, 0.1 M potassium phosphate buffer containing several known amounts of sodium chloride at 25 °C.

Table 1S. Specific retention factors of several common antibiotics determined for Aldrich humic acid at several flow rates and in pH 7.0, 0.10 M potassium phosphate buffer at 25 °C[a]

**Antibiotic**	**Specific retention factor (*k'*) and flow rate (mL/min)**			
	**0.25**	**0.10**	**0.05**	**Average *k'*[b]**
Sulfadiazine	0.31 (± 0.03)	0.28 (± 0.04)	0.25 (± 0.02)	0.28 (± 0.05)
Sulfamethoxazole	0.19 (± 0.03)	0.20 (± 0.03)	0.21 (± 0.02)	0.20 (± 0.05)
Lincomycin	4.11 (± 0.31)	3.05 (± 0.06)	3.33 (± 0.04)	3.50 (± 0.32)
Clarithromycin	6.36 (± 0.17)	6.21 (± 0.24)	8.30 (± 0.16)	6.96 (± 0.33)

[a]Specific retention factors were determined using 1.00 cm × 0.21 cm i.d. microcolumns. The numbers in parentheses are ± 1 S.D., as based on four injections and error propagation. The sample concentrations were 20 μM for sulfadiazine, sulfamethoxazole or lincomycin; and 10 μM for clarithromycin.

[b]This value is the average for the *k'* obtained at 0.25, 0.10, and 0.05 mL/min.

Table 2S. Total retention factors measured with microcolumns containing Aldrich humic acid (k_{Total}) or a control support ($k_{Control}$), and the corresponding specific retention factors (k') for several antibiotics, as obtained at pH 7.0 in 0.10 M potassium phosphate buffer and at various temperatures[a]

	Sulfadiazine			Sulfamethoxazole			Lincomycin			Clarithromycin		
Temp. (°C)	k_{Total}	$k_{Control}$	k'	k_{Total}	$k_{Control}$	k'	k_{Total}	$k_{Control}$	k'	k_{Total}	$k_{Control}$	k'
10	0.82 (± 0.02)	0.35 (± 0.02)	0.47 (± 0.02)	0.82 (± 0.02)	0.49 (± 0.02)	0.33 (± 0.03)	3.59 (± 0.08)	0.29 (± 0.01)	3.30 (± 0.08)	20.84 (± 0.22)	12.32 (± 0.08)	8.52 (± 0.24)
20	0.62 (± 0.02)	0.29 (± 0.02)	0.32 (± 0.03)	0.61 (± 0.01)	0.38 (± 0.02)	0.22 (± 0.03)	3.47 (± 0.04)	0.40 (± 0.03)	3.07 (± 0.05)	16.43 (± 0.12)	9.57 (± 0.16)	6.86 (± 0.20)
25	0.51 (± 0.03)	0.24 (± 0.03)	0.28 (± 0.04)	0.53 (± 0.02)	0.33 (± 0.03)	0.20 (± 0.03)	3.40 (± 0.05)	0.35 (± 0.03)	3.05 (± 0.06)	14.13 (± 0.16)	7.92 (± 0.18)	6.21 (± 0.24)
30	0.44 (± 0.01)	0.21 (± 0.02)	0.23 (± 0.03)	0.44 (± 0.04)	0.28 (± 0.03)	0.17 (± 0.04)	3.43 (± 0.15)	0.39 (± 0.02)	3.03 (± 0.15)	11.92 (± 0.09)	6.94 (± 0.11)	4.97 (± 0.14)
37	0.39 (± 0.01)	0.21 (± 0.02)	0.18 (± 0.02)	0.36 (± 0.02)	0.23 (± 0.02)	0.13 (± 0.03)	3.42 (± 0.03)	0.41 (± 0.02)	3.01 (± 0.04)	10.21 (± 0.09)	5.80 (± 0.09)	4.41 (± 0.13)
40	0.34 (± 0.01)	0.16 (± 0.02)	0.18 (± 0.03)	0.33 (± 0.01)	0.21 (± 0.02)	0.12 (± 0.02)	3.27 (± 0.08)	0.35 (± 0.02)	2.91 (± 0.09)	9.50 (± 0.05)	5.20 (± 0.10)	4.30 (± 0.11)
45	0.30 (± 0.02)	0.14 (± 0.02)	0.15 (± 0.03)	0.28 (± 0.02)	0.18 (± 0.02)	0.10 (± 0.03)	3.22 (± 0.04)	0.36 (± 0.02)	2.86 (± 0.04)	8.68 (± 0.08)	4.53 (± 0.06)	4.15 (± 0.10)

[a]These data were generated utilizing 1.00 cm × 0.21 cm i.d. microcolumns at 0.10 mL/min. The values in parentheses are ± 1 S.D. (n = 4), as determined from replicate injections and error propagation.

Table 3S. Distribution equilibrium constants (K_D) and global affinity constants (nK'_a) determined for Aldrich humic acid with several antibiotics in pH 7.0, 0.10 M potassium phosphate and at various temperatures[a]

Temperature	Sulfadiazine		Sulfamethoxazole		Lincomycin		Clarithromycin	
(°C)	K_D (× 10^1 L/kg)[b]	nK'_a (×10^3 L/mol)[c]	K_D (× 10^1 L/kg)[b]	nK'_a (× 10^3 L/mol)[c]	K_D (× 10^2 L/kg)[b]	nK'_a (× 10^4 L/mol)[c]	K_D (× 10^2 L/kg)[b]	nK'_a (× 10^4 L/mol)[c]
10	5.05 (± 0.27)	1.77 (± 0.09)	3.58 (± 0.27)	1.25 (± 0.10)	3.55 (± 0.09)	1.24 (± 0.03)	9.17 (± 0.26)	3.21 (± 0.09)
20	3.47 (± 0.31)	1.21 (± 0.11)	2.41 (± 0.28)	0.84 (± 0.10)	3.31 (± 0.06)	1.16 (± 0.02)	7.38 (± 0.21)	2.58 (± 0.07)
25	2.98 (± 0.25)	1.04 (± 0.09)	2.16 (± 0.24)	0.76 (± 0.08)	3.28 (± 0.06)	1.15 (± 0.02)	6.68 (± 0.30)	2.34 (± 0.09)
30	2.44 (± 0.29)	0.85 (± 0.10)	1.80 (± 0.37)	0.63 (± 0.13)	3.26 (± 0.02)	1.14 (± 0.06)	5.35 (± 0.15)	1.87 (± 0.05)
37	1.94 (± 0.23)	0.68 (± 0.79)	1.43 (± 0.27)	0.50 (± 0.09)	3.24 (± 0.04)	1.13 (± 0.02)	4.75 (± 0.13)	1.66 (± 0.05)
40	1.95 (± 0.29)	0.56 (± 0.10)	1.31 (± 0.25)	0.46 (± 0.09)	3.13 (± 0.10)	1.10 (± 0.03)	4.62 (± 0.12)	1.62 (± 0.04)
45	1.65 (± 0.31)	0.58 (± 0.11)	1.08 (± 0.22)	0.38 (± 0.08)	3.08 (± 0.04)	1.08 (± 0.01)	4.46 (± 0.10)	1.56 (± 0.04)

[a]The values in the parentheses are ± 1 S.D., as based on replicate sample injections (n = 4) and error propagation.

[b]The value of K_D was calculated by using the k' for an antibiotic at a given temperature (see Table 2S), the humic acid content of the support (20.7 mg/g silica) and the support's known packing density (0.45 mg/mL).

[c]The nK'_a values were determined by combining K_D with an average molar mass of 35,000 g/mol for Aldrich humic acid [1S].

Table 4S. Total retention factors measured with microcolumns containing Aldrich humic acid (k_{Total}) or a control support ($k_{Control}$), and the corresponding specific retention factors (k') for several antibiotics, as obtained at 25 °C and in 0.10 M potassium phosphate solutions at various pH values[a]

	Sulfadiazine			Sulfamethoxazole			Lincomycin			Clarithromycin		
pH	k_{Total}	$k_{Control}$	k'	k_{Total}	$k_{Control}$	k'	k_{Total}	$k_{Control}$	k'	k_{Total}	$k_{Control}$	k'
3.0	1.81 (± 0.02)	0.76 (± 0.01)	1.04 (± 0.02)	4.38 (± 0.02)	2.64 (± 0.02)	1.75 (± 0.03)	1.21 (± 0.02)	0.16 (± 0.01)	1.05 (± 0.02)	5.12 (± 0.05)	0.58 (± 0.01)	4.54 (± 0.05)
4.0	1.68 (± 0.02)	0.97 (± 0.01)	0.71 (± 0.04)	4.47 (± 0.09)	3.01 (± 0.03)	1.46 (± 0.10)	3.67 (± 0.20)	1.40 (± 0.02)	2.27 (± 0.20)	4.93 (± 0.10)	1.47 (± 0.02)	3.46 (± 0.10)
5.0	1.69 (± 0.03)	0.95 (± 0.02)	0.74 (± 0.04)	3.80 (± 0.05)	1.99 (± 0.04)	1.81 (± 0.06)	2.82 (± 0.04)	0.38 (± 0.02)	2.43 (± 0.04)	4.16 (± 0.07)	1.27 (± 0.02)	2.89 (± 0.08)
6.0	1.34 (± 0.02)	0.56 (± 0.02)	0.78 (± 0.03)	1.95 (± 0.03)	0.95 (± 0.02)	1.01 (± 0.04)	3.65 (± 0.04)	0.35 (± 0.02)	3.30 (± 0.05)	5.93 (± 0.06)	1.24 (± 0.02)	4.69 (± 0.07)
7.0	0.50 (± 0.01)	0.23 (± 0.02)	0.27 (± 0.02)	0.45 (± 0.01)	0.30 (± 0.02)	0.15 (± 0.02)	3.73 (± 0.10)	0.39 (± 0.02)	3.34 (± 0.10)	12.73 (± 0.12)	7.94 (± 0.09)	4.79 (± 0.15)
8.0	0.12 (± 0.01)	0.08 (± 0.02)	0.04 (± 0.02)	0.18 (± 0.02)	0.13 (± 0.02)	0.05 (± 0.02)	3.15 (± 0.13)	0.62 (± 0.02)	2.53 (± 0.13)	5.12 (± 0.05)	0.58 (± 0.01)	4.54 (± 0.05)

[a]These data were generated utilizing 1.00 cm × 0.21 cm i.d. microcolumns at 0.10 mL/min. The values in parentheses are ± 1 S.D. (n = 4), as determined from replicate injections and error propagation.

Table 5S. Total retention factors measured with microcolumns containing Aldrich humic acid (k_{Total}) or a control support ($k_{Control}$), and the corresponding specific retention factors (k') for several antibiotics at 25 °C and in pH 7.0, 0.10 M potassium phosphate buffer containing various added amounts of sodium chloride (NaCl)[a]

	Sulfadiazine			Sulfamethoxazole			Lincomycin			Clarithromycin		
**NaCl (M)**	k_{Total}	$k_{Control}$	k'	k_{Total}	$k_{Control}$	k'	k_{Total}	$k_{Control}$	k'	k_{Total}	$k_{Control}$	k'
0.00	0.51 (± 0.03)	0.24 (± 0.03)	0.28 (± 0.04)	0.53 (± 0.02)	0.33 (± 0.03)	0.20 (± 0.03)	3.40 (± 0.05)	0.35 (± 0.03)	3.05 (± 0.06)	16.13 (± 0.16)	7.92 (± 0.18)	8.21 (± 0.24)
0.10	0.51 (± 0.02)	0.29 (± 0.01)	0.21 (± 0.02)	0.52 (± 0.02)	0.40 (± 0.01)	0.11 (± 0.02)	2.24 (± 0.04)	0.38 (± 0.01)	1.86 (± 0.05)	16.69 (± 0.26)	9.07 (± 0.03)	7.62 (± 0.26)
0.20	0.57 (± 0.01)	0.35 (± 0.02)	0.22 (± 0.02)	0.61 (± 0.01)	0.45 (± 0.02)	0.16 (± 0.02)	2.08 (± 0.03)	0.33 (± 0.02)	1.75 (± 0.03)	16.68 (± 0.03)	9.52 (± 0.13)	7.17 (± 0.13)
0.30	0.58 (± 0.01)	0.36 (± 0.02)	0.22 (± 0.02)	0.66 (± 0.01)	0.50 (± 0.02)	0.16 (± 0.03)	2.14 (± 0.05)	0.37 (± 0.04)	1.77 (± 0.07)	17.01 (± 0.04)	10.06 (± 0.08)	6.96 (± 0.09)
0.40	0.60 (± 0.02)	0.38 (± 0.02)	0.22 (± 0.03)	0.70 (± 0.02)	0.54 (± 0.02)	0.16 (± 0.03)	1.82 (± 0.08)	0.32 (± 0.02)	1.50 (± 0.08)	15.23 (± 0.07)	10.58 (± 0.08)	5.65 (± 0.11)

[a]These data were generated at 0.10 mL/min on 1.00 cm × 0.21 cm i.d. microcolumns. The numbers in parentheses represent ± 1 S.D. (n = 4), as determined from replicate injections and error propagation.

Determination of fractions of acid-base forms and net charges of antibiotics vs pH

The plots shown in Figure 6 in the main body of the text present the predicted fractions of the various acid-base species expected for the antibiotics examined in this study as a function of pH. These plots were prepared by using the known pK_a values for these compounds, as based on a general approach described for estimating such fractions in other acid-base systems [2S,5S].

One case that may be present is a compound that contains a single acid/base group, resulting in two possible acid-base forms that may be present as the pH is varied. This situation can be described with a single acid dissociation constant (K_a) and its corresponding pK_a value, where pK_a = -log(K_a) [5S]. An example is clarithromycin, which contains one ionizable group and two possible acid-base forms: C^+, a singly-charged cation (the protonated conjugated acid form of the tertiary amine), and N, a neutral uncharged form (the unprotonated base form of the tertiary amine). The fractions present for each of these forms at a given pH (denoted below as f_{C^+} and f_N, respectively) can be calculated using the following formulas.

$$f_{C^+} = \frac{[H^+]}{K_a + [H^+]} \quad \text{(S4)}$$

$$f_N = \frac{K_a}{K_a + [H^+]} \quad \text{(S5)}$$

The plots for each of these fractions as a function of a pH (or $[H^+]$) were generated using these equations along with the K_a or pK_a values for each compound, and the relationship pH = -log$[H^+]$ or $[H^+] = 10^{-pH}$ [2S,5S].

A similar approach can be applied to chemicals with two sets of acid/base groups and three possible acid-base forms. Such systems have two pK_a values, as is the case for sulfadiazine, sulfamethoxazole, and lincomycin. For these antibiotics, the following acid-base forms can be present: C^+, a singly-charged cation; N or $Z^{\pm}$, a neutral form and/or zwitterion; and A^-, a singly-

charged anion. The fractions present for each of these forms at a given pH can be determined from the following relationships [2S,5S].

$$f_{\mathrm{C}^+} = \frac{[\mathrm{H}^+]^2}{[\mathrm{H}^+]^2 + K_{\mathrm{a1}}[\mathrm{H}^+] + K_{\mathrm{a1}}K_{\mathrm{a2}}} \quad \text{(S6)}$$

$$f_{\mathrm{N/Z}^\pm} = \frac{K_{\mathrm{a1}}[\mathrm{H}^+]}{[\mathrm{H}^+]^2 + K_{\mathrm{a1}}[\mathrm{H}^+] + K_{\mathrm{a1}}K_{\mathrm{a2}}} \quad \text{(S7)}$$

$$f_{\mathrm{A}^-} = \frac{K_{\mathrm{a1}}K_{\mathrm{a2}}}{[\mathrm{H}^+]^2 + K_{\mathrm{a1}}[\mathrm{H}^+] + K_{\mathrm{a1}}K_{\mathrm{a2}}} \quad \text{(S8)}$$

Examples of plots that use these equations to determine the fractions of these acid-base species for sulfadiazine, sulfamethoxazole, and lincomycin as a function of pH are provided in Figure 6 in the main body of the text.

Once the fraction of each acid-base species has been determined for a given compound, the net charge of that compound at a given pH can also be calculated, as shown in Figures 6 and Figure 7 in the main body of this paper. The net charge can be found by determining the sum of the fractions for each acid-base form multiplied by the charge for each form [2S,5S]. For instance, the net charge for a compound with one K_a value, such as clarithromycin, can be calculated as follows.

$$\text{Net charge} = [\{(+1) \cdot f_{\mathrm{C}^+}\} + \{(0) \cdot f_{\mathrm{N}}\}] \quad \text{(S9)}$$

For sulfadiazine, sulfamethoxazole, and lincomycin, each of which has two K_a values, the net charge can be determined from the equation given below.

$$\text{Net charge} = [\{(+1) \cdot f_{\mathrm{C}^+}\} + \{(0) \cdot f_{\mathrm{N/Z}^\pm}\} + \{(-1) \cdot f_{\mathrm{A}^-}\}] \quad \text{(S10)}$$

The functional groups responsible for the acid-base forms for the tested antibiotics are shown in the compound structures in Figure 1 of the main paper. For the sulfonamides (i.e., sulfadiazine and sulfamethoxazole), a sulfonyl group is located between an amine and an aniline group, resulting in a basic amine group ($-NH_2$) and an acidic amide group (-NH-). These groups allow these sulfonamides to form zwitterions ($SDZ^{\pm}$ or $SMX^{\pm}$), which are the predominant acid-base forms when the pH is between their pK_{a1} and pK_{a2} values (~pH 1.6/2.1-5.7/6.5). At pH values below pK_{a1} (1.6-2.1), the primary form of these antibiotics is a singly-charged cation (SDZ^{+} or SMX^{+}), while at pH values above pK_{a2} (5.7-6.5), the main form is a singly-charged anion (SDZ^{-} or SMX^{-}) [6S].

Lincomycin has three primary functional groups: a pyranose ring whose hydroxyl groups have pK_a values > 12.37; an amide group, with pK_a ~ -0.2; and a pyrrolidine ring, with pK_a ~ 7.97. For this antibiotic, when the pH falls below 7.97 the dominant acid-base form for this antibiotic is a singly-charged cation (LIN^{+}); between pH 7.97 and 12.37, a zwitterion ($LIN^{\pm}$) is predominant; and above pH 12.37, a singly-charged anion (LIN^{-}) is the main form [7S].

Clarithromycin is a 14-membered lactone ring with two deoxy sugars. The ionizable region in this structure is the dimethyl group on one of the deoxy sugars (pK_a, 8.99) [8S,9S]. Thus, the main acid-base form for clarithromycin is a singly-charged cation (CLA^{+}) at a pH less than about 9.0. The dominant species above this pH is the neutral form of this antibiotic (CLA).

Characterization of humic acid by size-exclusion chromatography

The molar mass and size of the Aldrich humic acid were examined by size-exclusion chromatography (SEC) [10S]. The HPLC system used for this purpose consisted of a UV-2075 absorbance detector, a PU-2080 pump, an AS-2057 Plus autosampler, and a DG-2080-54 degasser from JASCO (Easton, MD, USA). The SEC column used for this analysis was a BioSep SEC-s2000 column (5 µm, 145 Å, 300 mm × 7.8 mm I.D.) from Phenomenex (Torrance, CA), which was connected to two GFC 2000 guard cartridges (4 mm × 3 mm I.D.) that were packed the same support as the longer SEC analytical column. Prior to the sample injections, the columns and HPLC system were equilibrated with a mobile phase that consisted of pH 7,0, 0.10 M potassium phosphate buffer that was applied at 1.00 mL/min. After the system equilibration, the SEC column was calibrated using a set of protein standards with known molar masses and measured hydrodynamic diameters: immunoglobulin G (IgG, from human serum; purity ≥95%; product I4506), human serum albumin (HSA; ≥ 96%; product A1887), alpha$_1$-acid glycoprotein (AGP, from human plasma; ≥ 99%; product G9885), and myoglobin (from horse heart; ≥ 90%; product T-1882), which were all purchased from Sigma Aldrich (St. Louis, MO, USA).

The protein standards were each prepared at a concentration of 0.20 mg/mL in the same pH 7.0, 0.10 M phosphate buffer that was used as the mobile phase and were applied to the SEC column at 1.00 mL/min using a 20 µl injection volume. All these samples were analyzed in triplicate and filtered through 0.2 µm, 13 mm Acrodisc nylon syringe filter (Waters, Milford, MA, USA) before to their injection. The SEC column was calibrated with these standards at room temperature (22 °C) and the proteins were monitored through their absorbance at 280 nm. The SEC results obtained for the protein standards are summarized in Table 6S.

Following calibration of the SEC column, 20 µL injections of 0.10 mg/mL Aldrich humic acid were made under the same conditions, with elution of the humic acid now being monitored at 254 nm [10S-12S]. The Aldrich humic acid samples were prepared by first dissolving this humic acid preparation into pH 11.0, 0.10 M potassium phosphate buffer, followed by the gradual lowering of the pH for this solution to 7.0 through the addition of pH 2.5, 0.10 potassium phosphate buffer [12S-15S]. The final concentration of the humic acid was then adjusted to 0.10 mg/mL through its combination with additional pH 7.0, 0.10 M potassium phosphate buffer, with this sample solution then being passed through a 0.2 µm nylon filter before analysis on the SEC column. All chromatograms obtained from the HPLC system were analyzed using PeakFit 4.12 software (Jandel Scientific Software, San Rafael, CA, USA) employing a progressive linear baseline function.

The hydrodynamic diameters of the protein standards - IgG, HSA, AGP, and myoglobin - were determined using dynamic light scattering (DLS) with a ZetaStar I instrument (Wyatt, Santa Barbara, CA, USA). The measurements were carried out at 25 °C in a quartz cuvette and using a sample volume of 50 µL. During these measurements, each protein standard was prepared at a concentration of 0.05 mg/mL in pH 7.0, 0.10 M phosphate buffer, and filtered through a 0.2 µm nylon filter prior to analysis, in the same manner as done for SEC. The DLS measurements were obtained using a 785 nm laser and a 90° scattering angle, producing an average normalized scattering intensity of approximately 95.4 kilo counts/s. All measurements were performed in triplicate, and data processing was conducted using Dynamics Touch software v 2.0.1.164 from Wyatt. The results of the DLS measurements with the protein standards are also summarized in Table 6S.

Table 6S. The retention times and hydrodynamic diameters of standard proteins, as obtained by SEC and DLS[a]

Protein	Molar mass (kDa)	Retention time (min)	Hydrodynamic diameter (nm)
IgG	150	6.59 (± 0.01)	16.6 (± 0.2)
HSA	66.5	7.43 (± 0.01)	11.4 (± 0.8)
AGP	43	7.68 (± 0.01)	8.2 (± 0.2)
Myoglobin	16.7	8.94 (± 0.01)	3.4 (± 0.2)

[a]The conditions used in these experiments are given in the text. The molar masses were obtained from the literature [16S-19S]. The listed retention times were corrected for the system void time, which was 0.26 (± 0.01) min at 1.0 mL/min and 25 °C. The values in parentheses represent a range of ± 1 S.D. (n = 3).

A typical chromatogram that was obtained for the commercial preparation of Aldrich humic acid that was used in this study is shown in Figure 1S. Similar results were obtained for a different batch of this material that was obtained later from the same supplier. The chromatogram contained materials that spanned the entire calibration range of molar masses and hydrodynamic diameters for the SEC system. The distribution of this profile was asymmetric, including a group of high mass and larger species with elution times that ranged from roughly 5.3 to 7.5 min, a set of intermediate species eluting between 7.5 and 9.5 min, and a group of lower mass species that eluted between 9.5 min and the upper end of the calibration range at the column void time. Based on a response using the absorbance of these species at 254 nm, these three regions of the profile made up approximately 11.5%, 35.0%, and 53.5%, respectively, of the overall area measured in the calibration range of the SEC column.

Calibration of the size-exclusion column with proteins that had known hydrodynamic diameters allowed use of these SEC results to obtain direct estimates of the size distribution of particles within the Aldrich humic acid preparation. For instance, the first region for the Aldrich humic acid profile in Figure 1S at 5.3-7.5 min represented hydrodynamic particle diameters of 9.6 nm or larger. The second region from 7.5-9.5 min corresponded to diameters of 9.6 to 2.4 nm, with the central time in this region of 8.5 min being equivalent to a diameter of 4.8 nm. The third region at times of 9.5 min to the void time represented particle diameters of 4.8 nm or less, with the peak maximum in this region at about 10.0 min being equivalent to a particle diameter of 1.7 nm. In each case, the relative precision of the size estimate was around ± 26-33% (± 1 RSD).

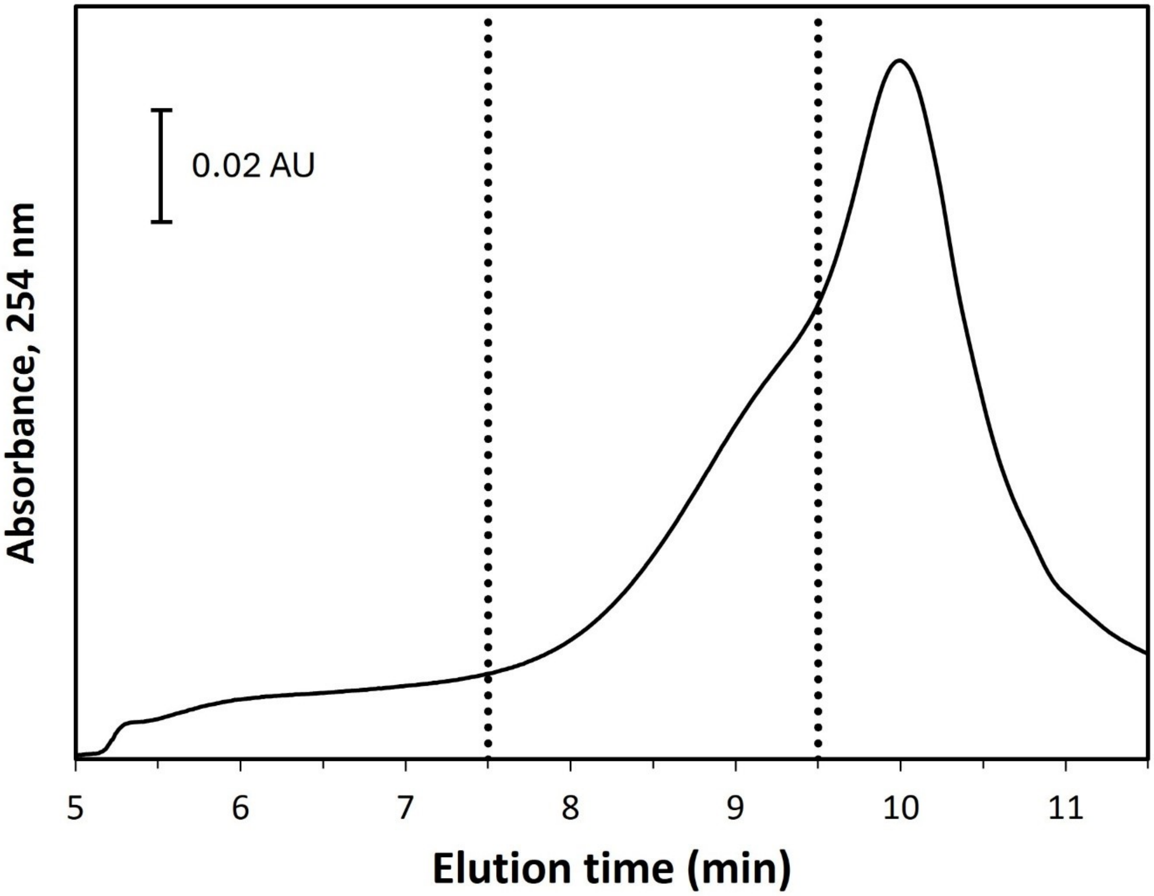

Figure 1S. SEC results obtained at 1.0 mL/min for the commercial sample of Aldrich humic acid that was used in this study and over the size-exclusion range of this system. The elution times have been corrected for the void time of the system with no column present (0.26 min). All other conditions are given in the text.

The molar mass for the Aldrich humic acid in these regions were also estimated from the SEC data in Figure 1S. This required considering the difference in densities between proteins and humic acid, as would affect the mass of a given size particle for each of these agents. The proteins that were used as calibration standards had densities of 1.33-1.44 g/mL (average, 1.38 g/mL) [20S-22S]. The density of humic acid tends to be larger, with a similar type of humic acid to that used in this work (i.e., Leonardite humic acid) having a reported particle density of 1.88 g/mL [23S], or a value 1.36-times higher than the average density of the protein standards. Using this proportionality factor, the first region for the profile in Figure 1S (5.3-7.5 min) represented humic acid particles with molar masses of roughly 60 kDa or larger. The second region (7.5-9.5 min) corresponded to molar masses of 9.3 to 60 kDa, and the third region (9.5 min to the void time) represented molar masses of 9.3 kDa or less. The relative precisions of these estimates of molar masses were in the range of ± 32-47% (± 1 RSD).

The average molar mass of 35 kDa that was used in Table 1 and the main body of the paper corresponded to the middle of the second and central region of the size distribution for Aldrich humic acid. For instance, the central time of 8.5 min in this region was equivalent to an estimated molar mass of 32 (± 13) kDa. As is indicated in the main body of the paper, the use of either higher or lower estimates for the average molar mass to calculate the association equilibrium constants (K_a) in Table 1 would result in proportionally higher or lower values for this term. However, use of a different average molar mass would not affect the results reported for the distribution equilibrium constant (K_D), as this term does not require knowledge of the molar mass for the binding agent.

Characterization of support porosity for entrapment-based immobilization

The elution times of HSA (molar mass, 66.5 kDa) and sodium nitrate were both determined using 20 µL injections for 10 µM solutions of these non-retained solutes. These injections were made on a 1.0 cm × 2.1 mm i.d. control column that was prepared using Nucleosil Si-300 that had been taken through the entire entrapment process but with no soluble humic acid being present. Similar injections were made with no column present in the system, as utilized to correct for the system void volume. Elution of the HSA and sodium nitrate was monitored at 280 nm or 205 nm, respectively. These samples were injected in triplicate or quadruplicate at 0.50 mL/min and 37 °C in the presence of pH 7.4, 0.067 M potassium phosphate buffer. The same pH 7.4 buffer was used to prepare the samples used for these injections.

Similar measurements of elution times were conducted with 20 µL injections of 20 µM sodium nitrate made on 2.0 cm × 2.1 mm i.d. columns containing diol-bonded silica, columns containing entrapped humic acid, and a control column that was prepared by entrapment but without the addition of humic acid. Each of these supports was again made using Nucleosil Si-300 as the starting material. These injections were performed in triplicate at 0.50 mL/min and 25 °C in the presence of pH 7.0, 0.10 M potassium phosphate buffer as the mobile phase. The same buffer was used to prepare the sample solutions. In all these studies, the chromatograms and central moments of the resulting peaks were analyzed using PeakFit v4.12 software (Jandel Scientific, San Rafael, CA, USA).

References

[1S] S. Iftekhar, S. Poddar, M. Rauhauser, D.D. Snow, D.S. Hage, Preparation of entrapment-based microcolumns for analysis of drug-humic acid interactions by high-performance affinity chromatography, Anal. Chim. Acta 1239 (2023) 340629.

[2S] S. Sharmeen, I. Kyei, A. Hatch, K. Suh, S. Poddar, S. Iftekhar, D. D. Snow, D.S. Hage, Analysis of interactions between pharmaceuticals and humic acid: characterization using entrapment and high-performance affinity microcolumns, J. Chromatogr. A 1737 (2024) 465427.

[3S] J. Chen, D.S. Hage, Quantitative affinity chromatography: practical aspects, in: D.S. Hage, (Ed.), Handbook of Affinity Chromatography, Taylor & Francis, Boca Raton, 2005, pp. 595–627.

[4S] J. Vargas-Badilla, S. Poddar, S. Azaria, C. Zhang, D.S. Hage, Optimization of protein entrapment in affinity microcolumns using hydrazide-activated silica and glycogen as a capping agent, J. Chromatogr. B 1121 (2019) 1–8.

[5S] D.S. Hage, J. D. Carr, Analytical Chemistry and Quantitative Analysis, Prentice Hall, Hoboken, 2011.

[6S] E. Çalişkan, S. Göktürk, Adsorption characteristics of sulfamethoxazole and metronidazole on activated carbon, Sep. Sci. Technol. 45 (2010) 244–255.

[7S] A. Mehrtens, T. Licha, V. Burke, Occurrence, effects and behaviour of the antibiotic lincomycin in the agricultural and aquatic environment – a review, Sci. Total Environ. 778 (2021) 146306.

[8S] I. Christl, M. Ruiz, J.R. Schmidt, J.A. Pedersen, Clarithromycin and tetracycline binding to soil humic acid in the absence and presence of calcium, Environ. Sci. Technol. 50 (2016)

9933−9942.

[9S] Y. Nakagawa, S. Itai, T. Yoshida, T. Nagai, Physiochemical properties and stability in the acidic solution of a new macrolide antibiotic, clarithromycin, in comparison with erythromycin, Chem. Pharm. Bull. 40 (1992) 725–728.

[10S] J. Song, W. Huang, P. Peng, B. Xiao, Y. Ma, Humic acid molecular weight estimation by high-performance size-exclusion chromatography with ultraviolet absorbance detection and refractive index detection, Soil Sci. Soc. Am. J. 74 (2010) 2013–2020.

[11S] R.S. Summers, P.K. Cornel, P.V. Roberts, Molecular size distribution and spectroscopic characterization of humic substances, Sci. Total Environ. 62 (1987) 27–37.

[12S] L.A. Tercero Espinoza, E. ter Haseborg, M. Weber, F.H. Frimmel, Investigation of the photocatalytic degradation of brown water natural organic matter by size exclusion chromatography, Appl. Catalysis B: Environ. 87 (2009) 56–62.

[13S] L. Li, Z. Zhao, W. Huang, P. Peng, G. Sheng, J. Fu, Characterization of humic acids fractionated by ultrafiltration, Org. Geochem. 35 (2004) 1025–1037.

[14S] A. Baglieri, D. Vindrola, M. Gennari, M. Negre, Chemical and spectroscopic characterization of insoluble and soluble humic acid fractions at different pH values, Chem. Biol. Tech. Agri. 1 (2014) 9.

[15S] M. Klučáková, M. Pekař, Solubility and dissociation of lignitic humic acids in water suspension, Coll. Surf. A: Physicochem. Eng. Aspects 252 (2005) 157–163.

[16S] S.R. Beeram, C. Zhang, K. Suh, W.A. Clarke, D.S. Hage, Characterization of drug binding with alpha$_1$-acid glycoprotein in clinical samples using ultrafast affinity extraction. J. Chromatogr. A 1649 (2021) 462240.

[17S] A.J. Jackson, E.M. Karle, D.S. Hage, Preparation of high-capacity supports containing protein G immobilized to porous silica. Anal. Biochem. 406 (2010) 235–237.

[18S] J. Zaia, R.S. Annan, K. Biemann, The correct molecular weight of myoglobin, a common calibrant for mass spectrometry. Rapid Com. Mass Spec. 6 (1992) 32–36.

[19S] S.T. Ovbude, P. Tao, Z. Li, D.S. Hage, High-Performance affinity chromatographic studies of repaglinide and nateglinide interactions with normal and glyoxal- or methylglyoxal-modified human albumin serum. J. Pharm. Biomed. Anal. 201 (2021) 114097.

[20S] M.L. Quillin, B.W. Matthews, Accurate calculation of the density of proteins, Acta Cryst. D56 (2000) 791–794.

[21S] J. Voros, The density and refractive index of adsorbing protein layers, Biophys. J. 87 (2004) 553–561.

[22S] J. Tsai, R. Taylor, C. Chothia, M. Gerstein, The packing density in proteins: Standard radii and volumes, J. Mol. Biol. 290 (1999) 253–266.

[23S] B. Kandra, A. Tall, J. Vitkova, M. Prochazka, P. Surda, Effect of humic amendment on selected hydrophysical properties of sandy and clayey soils, Water 16 (2024) 1338.
